# OsGRF6‐OsYUCCA1/OsWRKY82 Signaling Cascade Upgrade Grain Yield and Bacterial Blight Resistance in Rice

**DOI:** 10.1002/advs.202407733

**Published:** 2024-10-23

**Authors:** Huanran Yuan, Mingxing Cheng, Fengfeng Fan, Xingfei Zheng, Ruihua Wang, Fengfeng Si, Xiong Luo, Nengwu Li, Shaoqing Li

**Affiliations:** ^1^ State Key Laboratory of Hybrid Rice Key Laboratory for Research and Utilization of Heterosis in Indica Rice of Ministry of Agriculture Engineering Research Center for Plant Biotechnology and Germplasm Utilization of Ministry of Education College of Life Sciences Wuhan University Wuhan 430072 China; ^2^ Hubei Shizhen Laboratory Hubei University of Chinese Medicine Wuhan 430065 China; ^3^ Hubei Key Laboratory of Food Crop Germplasm and Genetic Improvement Food Crop Institute Hubei Academy of Agricultural Sciences Wuhan Hubei 430064 China

**Keywords:** Bacterial blight resistance, grain yield, OsGRF6, OsYUCCA1, OsWRKY82

## Abstract

As a major crop in the world, the sustainable development of rice is often severely restricted by bacterial blight. Breeding crops with resistance is an efficient way to control bacterial blight. However, enhancing resistance often incurs a fitness penalty, making it challenging to simultaneously increase bacterial blight resistance and yield potential. In this study, it is found that *OsGRF6*, besides being a high‐yield gene, can significantly improve rice bacterial blight resistance. Compared with wild‐type, the lesion lengths of transgenic material overexpressing *OsGRF6* are significantly reduced after inoculation with *Xanthomonas oryzae pv. oryzae* (*Xoo*). Furthermore, OsGRF6 can directly bind to the promoters of *OsYUCCA1* and *OsWRKY82*, upregulating their transcription and thereby increasing rice bacterial blight resistance and yield. Haplotypic analysis based on the promoter and genome sequence combined with evolutionary analysis revealed that *OsGRF6* is mainly comprised by the *OsGRF6^XI^* and *OsGRF6^GJ^* subtypes. The superior haplotype *OsGRF6* ^Hap4^ increased its transcriptional activity and contributed to bacterial blight resistance and rice yield. Together, this study provides theoretical support for further revealing the synergistic regulatory mechanism and genetic improvement of rice high yield and bacterial blight resistance, offering a new strategy for developing disease‐resistant cultivars.

## Graphical abstract

In this study, *OsGRF6* enhances rice grain yield by regulating *OsYUCCA1*, which modulates auxin metabolism and promotes secondary branch development. During bacterial blight infection, *OsGRF6* transcription increases, activating *OsWRKY82* and triggering disease‐responsive genes, H_2_O_2_ accumulation, and the JA pathway for improved resistance.

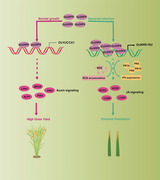

## 1 Introduction

Rice is the staple crop for more than half of the world's population, but its sustainable production is threatened by various pests and diseases.^[^ ^1^, ^2^, ^3^ ^]^ Diseases such as fungal blast caused by *Magnaporthe oryzae*, bacterial blight caused by *Xanthomonas oryzae* pv. *oryzae* (*Xoo*), and bacterial leaf streak caused by *Xanthomonas oryzae* pv. *oryzicola* (*Xoc*), can result in yield losses of up to 30% −70%.^[^ ^3^, ^4^, ^5^, ^6^ ^]^ To effectively control rice disease, major resistance (*R*) genes, which encode nucleotide‐binding leucine‐rich repeat immune receptors to activate effector‐triggered immunity (ETI), are utilized in many resistant rice cultivars.^[^ ^7^, ^8^, ^9^, ^10^, ^11^ ^]^ However, resistance conferred by single *R* genes is vulnerable to disease races that lack the corresponding avirulence effectors. Although stacking multiple *R* genes in a cultivar can expand the anti‐race spectrum, it often results in a yield penalty.^[^ ^12^, ^13^, ^14^ ^]^ Consequently, there is an urgent need to develop rice varieties with genes that provide non‐race‐specific resistance without compromising yield.

In the past decades, great many of growth or disease resistance related genes have been cloned. However, fewer genes have been identified that balance plant growth and immunity.^[^ ^15^, ^16^, ^17^ ^]^ For instance, *BRASSINAZOLE‐RESISTANT1* (*BZR1*), along with its target INTERACTING WITH IBH1 (HBI1) and interactor PHYTOCHROME INTERACTING 4 (PIF4), suppresses immunity during rapid plant growth.^[^ ^18^, ^19^, ^20^ ^]^ Conversely, genes such as atypical *DP‐E2F‐like 1* (*DEL1*), *TL1‐BINDING FACTOR 1* (*TBF1*) and *WRKY45* enhance pathogen resistance but inhibit plant growth.^[^ ^21^, ^22^, ^23^ ^]^ Breeding practices have significantly advanced the delicate balance between yield and immunity.^[^ ^3^, ^16^, ^24^ ^]^ The transcription factor *Ideal Plant Architecture 1* (*IPA1*) promotes yield and disease resistance by maintaining this balance. Under normal conditions, IPA1 binds to the promoter of *Dense and Erect Panicle 1* (*DEP1*), regulating its expression level and promoting plant growth and yield. Upon pathogen attack, IPA1 becomes phosphorylated at the DNA binding site and binds to the promoter of *WRKY45* to enhanced immunity to *M. oryzae*.^[^ ^16^ ^]^ OsUBC45, a ubiquitin‐conjugating enzyme, promotes disease resistance and yield in rice. Overexpression of *OsUBC45* increased rice yield by 10% to 15% and significantly enhanced resistance to blast disease and bacterial blight.^[^ ^3^ ^]^ *UMP1^R2115^*, a natural allele of proteasome maturation factor in rice, confers broad‐spectrum resistance to *M. oryzae*, *Rhizoctonia solani*, *Ustilaginoidea virens*, and *Xoo*.^[^ ^25^ ^]^ Introduction of *UMP1^R2115^* into a disease‐susceptible rice variety does not reduce rice yield while improving disease resistance.^[^ ^25^ ^]^ This underscores the pivotal role of certain genes in enhancing both plant yield and disease resistance. Therefore, illustrating the superior genes that could generate novel elite varieties with high yield and enhanced resistance, and developing the key technical strategy to achieve high yield and high resistance aggregation are big challenges in breeding science.

The growth‐regulating factor (GRF) gene family consists of plant‐specific transcription factors,^[^ ^26^, ^27^ ^]^ GRF proteins typically contain highly conserved QLQ and WRC domains, which function in protein‐protein interactions and DNA binding, respectively.^[^ ^28^ ^]^ Recent studies have underscored GRFs as pivotal transcription factors that regulate diverse aspects of plant growth and development, including roots, leaves, and floral organs.^[^ ^29^, ^30^ ^]^ In *Arabidopsis thaliana*, triple null mutants of *grf1/2/3* display smaller and narrower leaves, while overexpression of *AtGRF1* or *AtGRF2* leads to larger leaves and longer petioles.^[^ ^31^ ^]^ In rice, overexpression of *OsGRF3* and *OsGRF10* induces adventitious root formation at nodes due to abnormal meristematic activity.^[^ ^32^, ^33^ ^]^ Downregulation of *OsGRF1* in *rhd1* mutants leads to an earlier heading date.^[^ ^34^ ^]^ OsGRF4/GS2/GL2 interacts with OsGIF1–3 to increase grain size and weight.^[^ ^35^, ^36^, ^37^ ^]^ *OsGRF8*, is target of miR396, shows increased grain size and elongated panicles when the miR396 target site is disrupted.^[^ ^38^ ^]^ *OsGRF6* controls inflorescence architecture and grain yield, overexpression of *OsGRF6* enhances inflorescence branching and rice yield.^[^ ^39^ ^]^ It activates the expression of the *JMJD2* family jmjC gene 706 (*OsJMJ706*) and *crinkly4 receptor‐like kinase* (*OsCR4*) to regulate floret development.^[^ ^40^ ^]^ Moreover, *OsGRF6* is crucial for responding to both biotic and abiotic stresses, contributing to the balance between growth and disease resistance in rice,^[^ ^41^, ^42^, ^43^ ^]^ it targets *OsMYB3R*,^[^ ^41^, ^42^ ^]^ and *GA20‐oxidase 1* (*GA20ox 1*),^[^ ^43^ ^]^ enhancing salt and chilling tolerance, respectively.

Here, we report that *OsGRF6*, not only enhances rice yield but also confers resistance to bacterial blight. It directly targets downstream genes *OsYUCCA1* and *OsWRKY82* to promote secondary branching and enhance bacterial blight resistance, highlighting its dual role in improving both rice yield and immune response. This discovery may pave a way for designing molecular strategies aimed at developing high‐yield, disease‐resistant super rice varieties.

## 2 Results

### 2.1 *OsGRF6* Promotes Both Bacterial Blight Resistance and Grain Yield in Rice

**Figure 1:**
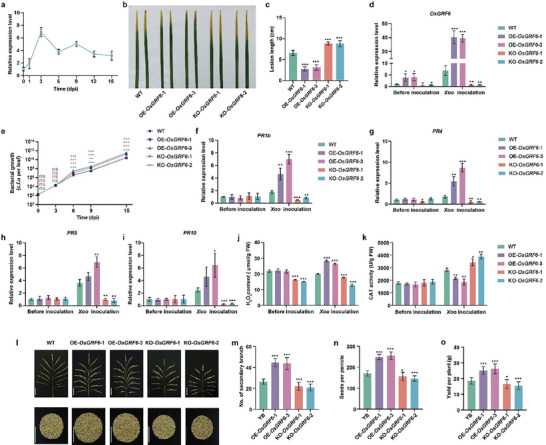
Identification of bacterial blight resistance and grain yield of *OsGRF6* transgenic lines. a) qRT–PCR analysis of the *OsGRF6* expression after *Xoo* PXO99 inoculation at 30 days of seedings. Data are mean ± s.d. (*n* = 3). b) Phenotype of the wild type (WT, YB), OE‐*OsGRF6* lines, and KO‐*OsGRF6* mutants after *Xoo* infection. The 30‐day‐old leaves of WT and *OsGRF6* transgenic lines were inoculated with the *Xoo* PXO99. Leaves were photographed at the 14th day post‐inoculation (dpi). c) Lesion lengths on *OsGRF6* transgenic lines after *Xoo* inoculation. Leaves were measured at 6 dpi. Data are mean ± s.d. (*n* = 5). d) Expression levels of *OsGRF6* were analysed in WT and *OsGRF6* transgenic lines were treated with or without *Xoo* inoculation. Leaves from 30‐day‐old plants were inoculated with Xoo for 6 days, then collected for qRT‐PCR analysis. Data are mean ± s.d. (*n* = 3). e) Growth of *Xoo*, indicated by the number of colony‐forming units (c.f.u.) per leaf for *OsGRF6* transgenic lines. The bacterial growth in WT and *OsGRF6* transgenic lines was calculated at 0, 3, 6, 9, and 15 dpi compared with the value at 0 day. Data are mean ± s.d. (*n* = 5). f‐i) Relative expression levels of *PR1b* (f), *PR4* (g), *PR5* (h), and *PR10* (i) in *OsGRF6* transgenic lines and in the WT. All leaves (30‐day‐old) were inoculated with the *Xoo* for 6 days. Data are mean ± s.d. (*n* = 3). j, k) Measurements of H_2_O_2_ content (H_2_O_2_, j), catalase activity (CAT, k). Measurements of H_2_O_2_ content and CAT activity of WT and *OsGRF6* transgenic lines were performed before and after inoculated with *Xoo* for 6 days. Data are mean ± s.d. (*n* = 3). l) Panicle and grains per plant of WT, OE‐*OsGRF6* lines, and KO‐*OsGRF6* mutants. Scale bar, 10 cm. m‐o) Statistical analysis of secondary branch number (m), seeds per panicle (n), and grain yield per plant (o) in WT, OE‐*OsGRF6* lines, and KO‐*OsGRF6* mutants. Different asterisks in a, c, d, e, f, g, h, i, j, k, m, n, and o indicate significant differences determined by Student's *t‐*test (*, *p* < 0.05; **, *p* < 0.01; ***, *p* < 0.001).

As a transcriptional factor, *OsGRF6* has been demonstrated enhancing rice grain yield and the fungal pathogen *M. oryzae* resistance,^[^ ^41^ ^]^ we still have limited understanding of how it regulates the secondary branch development and the immune response to bacterial blight infection. To address this, qRT‐PCR confirmed that the expression level of *OsGRF6* was enhanced at 3 days post‐inoculation (dpi) with the bacterial pathogen *Xoo* and remained high for at least 15 dpi (**Figure** **1**a). Transcriptome analysis of rice leaves revealed numerous differentially expressed genes (DEGs) associated with “response to biotic stimulus”, “response to endogenous stimulus”, and “response to oxygen‐containing compound” (Figure S1, Supporting Information), suggesting that *OsGRF6* plays an important role in plant bacterial blight resistance and immunity, and that *Xoo* might manipulate *OsGRF6* expression to enhance pathogenesis. To investigate the role of *OsGRF6* in the defense response, transgenic lines of OE‐*OsGRF6* and KO‐*OsGRF6* and their corresponding wild‐type (WT, YB) plants, were inoculated with the PXO99 of *Xoo* using the leaf‐clipping method. Lesions were significantly shorter in OE‐*OsGRF6* transgenic plants compared to WT (Figure 1b,c), and the expression of *OsGRF6* was significantly increased in *OsGRF6* overexpression transgenic materials, especially after *Xoo* infection (Figure 1d). Conversely, lesions were significantly longer in KO‐*OsGRF6* transgenic plants than in the WT plants (Figure 1b,c), demonstrating that *OsGRF6* positively regulated disease resistance against *Xoo*. Additionally, bacterial growth was distinctly lower in the OE‐*OsGRF6* transgenic rice than in WT plant and KO‐*OsGRF6* transgenic plants (Figure 1e). The transcript levels of the defense marker genes *PR1b*, *PR4*, *PR5*, and *PR10* were greater in OE‐*OsGRF6* transgenic plants than in WT plants, and lower in KO‐*OsGRF6* transgenic plants than in WT plants (Figure 1f–i), indicating that *OsGRF6* positively regulate defense responses.

Reactive oxygen species (ROS) are generally considered biomarkers of plant response to environmental stress.^[^ ^42^, ^44^ ^]^ Here, more H_2_O_2_ accumulated in the *OsGRF6* overexpression lines, but less in KO*‐OsGRF6* mutant than that in their corresponding WT when inoculated with the *Xoo* (Figure 1j). As expected, the ROS‐scavenging enzyme activities of catalase (CAT) and peroxidase (POD) were consistent with the changing trend of H_2_O_2_ accumulation, with decreased H_2_O_2_ and drastically enhanced activities of CAT and POD in KO‐*OsGRF6* transgenic lines (Figure 1k; Figure S2, Supporting Information). Previously, *OsGRF6* has been shown to increasing rice yield by promoting secondary branch development.^[^ ^39^ ^]^ With the increase of *OsGRF6* expression, the number of secondary branches, seeds per panicle, and grain yield per plant were significantly increased in OE‐*OsGRF6* transgenic lines, while the opposite phenotype was observed in KO‐*OsGRF6* transgenic lines (Figure 1l–o). Taken together, the results indicate that the activation of *OsGRF6* promotes both bacterial blight resistance and yield in rice.

### 2.2 *OsWRKY82* is a Key Target of *OsGRF6* Responding to Pathogen Infection

**Figure 2:**
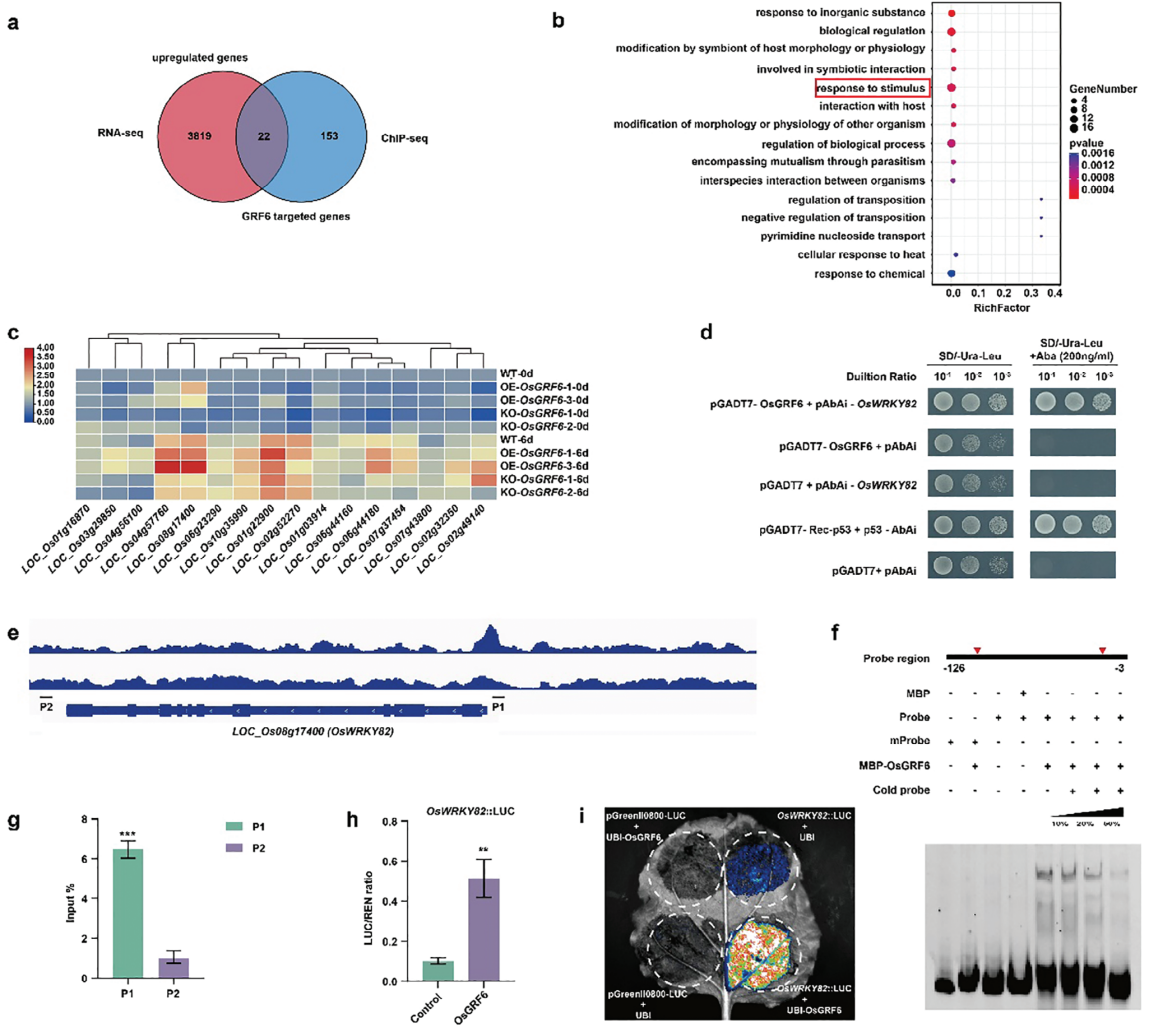
*Os*GRF6 targets *OsWRKY82* to regulate bacterial blight resistance in rice. a) Overlapping analysis of the OsGRF6 potential target genes via RNA‐seq and ChIP‐seq in rice. b) GO enrichment analysis of the overlapped genes in (a). The red box indicates signaling pathways responding to stimulus. c) Expression analysis of the 16 genes under the GO term of “responding to stimulus” with or without *Xoo* inoculation. d) Yeast one‐hybrid assay for OsGRF6 binding to the *OsWRKY82* promoter. Growth of yeast cells transformed with prey (pGADT7‐OsGRF6) and bait (pAbAi‐*OsWRKY82*), along with negative control (pGADT7 + pAbAi) and positive control (pGADT7‐Rec‐p53 + p53‐Abai), on selective medium with or without AbA (200 ng mL^−1^). e) ChIP‐seq showing the OsGRF6 binding peak to the *OsWRKY82* promoter. Black lines indicate probe loci (P1, binding sites; P2, used as negative control). f) EMSA analysis of OsGRF6 binding to *OsWRKY82* promoter. The mutated probe was generated by mutating all the CGC(G)A(C)G(A) motifs in the *OsWRKY82* promoter sequence from −3 to −126 to CAC(G)A(C)G(A). g) ChIP‐qPCR validation for the binding sites in (e). h, i) OsGRF6 activates transcription of *OsWRKY82* as shown by dual‐luciferase transcriptional activity assay in rice protoplasts (h) and tobacco (i). Different asterisks in g and h indicate significant differences determined by Student's *t‐*test (**, *p* < 0.01; ***, *p* < 0.001). Data are mean ± s.d. (*n* = 3).

To dissect the downstream signaling pathways regulated by the *OsGRF6* in response to bacterial blight infection, the transcriptomes of *Xoo*‐inoculated rice leaves from wild‐type and OE‐*OsGRF6* transgenic plants were analyzed. A total of 3841 upregulated genes were identified in the OE‐*OsGRF6* transgenic plants (Figure S3, Supporting Information). Combined analysis revealed an overlap of 22 genes between the upregulated genes identified by RNA‐seq and our previous ChIP‐seq data of OE‐*OsGRF6*,^[^ ^39^ ^]^ suggesting these are potential targets of *OsGRF6* in the immune response (**Figure** **2**a). GO analysis showed that 16 of these 22 genes were annotated as involved in the “response to stimulus” process (Figure 2b). qRT‐PCR analysis demonstrated that *LOC_Os04g57760* and *LOC_Os08g17400* were up‐regulated in the OE‐*OsGRF6* transgenic lines but down‐regulated in the KO‐*OsGRF6* transgenic plants, especially upon *Xoo* inoculation (Figure 2c). Further analysis revealed that *LOC_Os08g17400* encodes OsWRKY82, a protein involved in defense response,^[^ ^45^, ^46^ ^]^ whereas LOC_Os04g57760 encodes a protein with unknown function. These results suggest that *OsWRKY82* is a potential target of *OsGRF6* in response to bacterial blight resistance.

To further verify if *OsWRKY82* is a direct target of OsGRF6, a yeast one‐hybrid assay was conducted. Results showed that OsGRF6 could bind the *OsWRKY82* promoter sequence (Figure 2d), and a distinct binding peak was observed in the ChIP‐seq data (Figure 2e). Previous studies have shown that the binding motif of OsGRF6 proteins is CGC(G)A(C)G(A).^[^ ^39^ ^]^ EMSA combined with ChIP‐qPCR analysis revealed that OsGRF6 could bind to a DNA probe containing the CGC(G)A(C)G(A) element in the *OsWRKY82* promoter (Figure 2f,g). Moreover, a luciferase reporter assay in rice protoplasts and tobacco demonstrated that OsGRF6 activates the expression of *OsWRKY82* (Figure 2h), with a stronger fluorescence signal observed in tobacco leaves (Figure 2i). Collectively, these findings illustrate that OsGRF6 directly targets to *OsWRKY82* to activate its expression in response to bacterial blight resistance.

### 2.3 *OsWRKY82* Improves Rice Bacterial Blight Resistance

**Figure 3:**
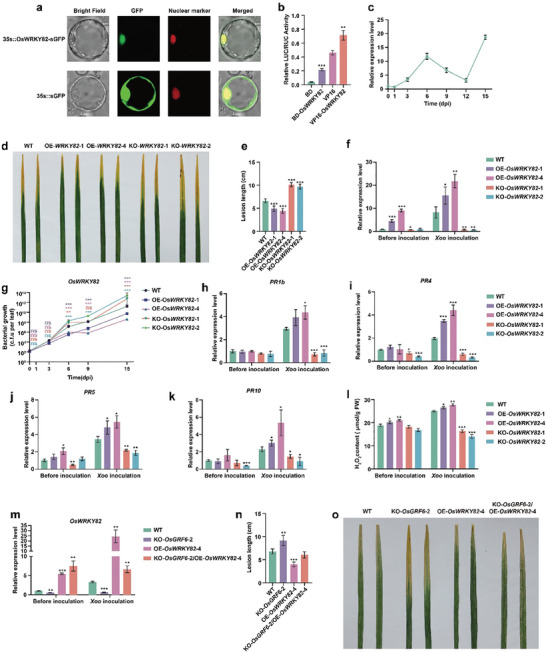
*OsWRKY82* positively regulates bacterial blight resistance in rice. a) Subcellular localization of OsWRKY82‐GFP fusion protein in rice protoplasts. D53–mCherry was used as a nuclear marker. Scale bars, 5 µm. b) Transcriptional activation assay demonstrated that OsWRKY82 displays active transcriptional activity in rice protoplasts. Data are mean ± s.d. (*n* = 3). c) qRT‐PCR analysis of *OsWRKY82* expression after *Xoo* inoculation at different time point. Data are mean ± s.d. (*n* = 3). d, e) Phenotypes and lesion lengths of *OsWRKY82* overexpression (OE‐*OsWRKY82*) and knockout (KO‐*OsWRKY82*) lines after *Xoo* inoculation. The 30‐day‐old leaves of WT and *OsWRKY82* transgenic lines were inoculated with the *Xoo* PXO99. The leaves were photographed and measured at 14 dpi. Data are mean ± s.d. (*n* = 5). f) Expression level of *OsWRKY82* in the WT and *OsWRKY82* transgenic lines with or without *Xoo* inoculation. The 30‐day‐old leaves were inoculated with the *Xoo* for 6 days and collected for qRT‐PCR analysis. Data are mean ± s.d. (*n* = 3). g) Growth of *Xoo* in leaves of the OE‐*OsWRKY82* lines and KO‐*OsWRKY82* mutants. The bacterial growth in WT and *OsWRKY82* transgenic lines were calculated at 0, 3, 6, 9, and 15 dpi compared with the value at 0 day. Data are mean ± s.d. (*n* = 3). h‐k) The relative expression level of *PR1b* (h), *PR4* (i), *PR5* (j), and *PR10* (k) in the *OsWRKY82* transgenic lines. All leaves (30‐day‐old) were inoculated with the *Xoo* for 6 days. Data are mean ± s.d. (*n* = 3). l) Measurements of H_2_O_2_ content in the *OsWRKY82* transgenic lines after *Xoo* inoculation for 6 days. Data are mean ± s.d. (*n* = 3). m) Expression level of *OsWRKY82* in the WT, KO‐*OsGRF6*‐2, OE‐*OsWRKY82*‐4 and KO‐*OsGRF6*‐2/OE‐*OsWRKY82*‐4 lines after *Xoo* inoculation for 6 days. Data are mean ± s.d. (*n* = 3). n, o) Phenotypes and lesion lengths of the WT, KO‐*OsGRF6*‐2, OE‐*OsWRKY82*‐4, and KO‐*OsGRF6*‐2/OE‐*OsWRKY82*‐4 lines after *Xoo* inoculation. The 30‐day‐old leaves were inoculated with the *Xoo* and the leaves were photographed and measured at 14 dpi. Data are mean ± s.d. (*n* = 5). Different asterisks in b, c, e, f, g, h, i, j, k, l, m, and n indicate significant differences determined by Student's *t‐*test (*, *p* < 0.05; **, *p* < 0.01; ***, *p* < 0.001).

Bioinformatics analysis revealed that OsWRKY82 encodes a transcription factor belonging to the WRKY family, characterized by two conserved WRKY domains (Figure S4, Supporting Information). Transcriptional activity assay and subcellular localization studies confirmed OsWRKY82's potential role as a transcriptional activator (**Figure** **3**a,b). Phylogenetic analysis revealed that OsWRKY82 is highly similar to several proteins within its subclade (Figure S5, Supporting Information), such as OsWRKY104, OsWRKY95, OsWRKY40, and OsWRKY64 that are known to respond to biotic and abiotic stresses^[^ ^47^, ^48^ ^]^ suggesting that may play a crucial role in coping with environmental stress. Consistent with this notion is that expression of the ubiquitously expressed *OsWRKY82* was progressively induced by the *Xoo* inoculation (Figure 3c; Figure S6, Supporting Information).

To validate the biological function of *OsWRKY82* in rice, *OsWRKY82* overexpression (OE‐*OsWRKY82*) and knockout mutants (KO‐*OsWRKY82*) were generated (Figures S7 and S8, Supporting Information). Phenotype analysis revealed that, compared to the WT, the *OsWRKY82* transgenic lines showed no significant differences in the number of secondary branches, seeds per panicle, and grain yield per plant (Figure S9, Supporting Information). When infected with bacterial blight, lesions were significantly shorter in OE‐*OsWRKY82* transgenic rice compared to WT and KO‐*OsWRKY82* plants inoculated with *Xoo* PXO99 (Figure 3d,e). Expression level of *OsWRKY82* was markedly elevated in OE‐*OsWRKY82* plants relative to WT, whereas significantly down‐regulated in KO‐*OsWRKY82* transgenic plants under *Xoo* inoculation (Figure 3f). Bacterial growth was lower in OE‐*OsWRKY82* plants at 6, 9, and 15 dpi, consistent with enhanced bacterial blight resistance (Figure 3g). Additionally, defense marker genes *PR1b*, *PR4*, *PR5*, and *PR10* were significantly upregulated in OE‐*OsWRKY82* transgenic plants (Figure 3h–k), accompanied by increased H_2_O_2_ accumulation (Figure 3l) and reduced activities of ROS‐scavenging enzymes CAT and POD (Figure S10, Supporting Information). As expected, these trends were reversed in KO‐*OsWRKY82* lines (Figure S10, Supporting Information). Together, these findings establish *OsWRKY82* as a positive regulator of rice disease resistance against bacterial blight. To explore the genetic interaction between *OsGRF6* and *OsWRKY82*, we generated a double transgenic line KO‐*OsGRF6*‐2/OE‐*OsWRKY82*‐4 by crossing KO‐*OsGRF6*‐2 with OE‐*OsWRKY82*‐4 lines (Figure 3m–o). Upon *Xoo* inoculation, lesion lengths were reduced in the KO‐*OsGRF6*‐2/OE‐*OsWRKY82*‐4 line compared to KO‐*OsGRF6*‐2 lines (Figure 3n,o), accompanied by upregulation of *OsWRKY82* expression, confirming *OsWRKY82* could rescue the bacterial blight resistance phenotype of *OsGRF6* (Figure 3m; Figure S11, Supporting Information). These results collectively indicate that *OsGRF6* activates *OsWRKY82* expression, thereby mitigating pathogen effects on rice development and growth.

### 2.4 *OsYUCCA1* is a Key Target of *OsGRF6* Regulating the Secondary Branch Development in Rice

**Figure 4:**
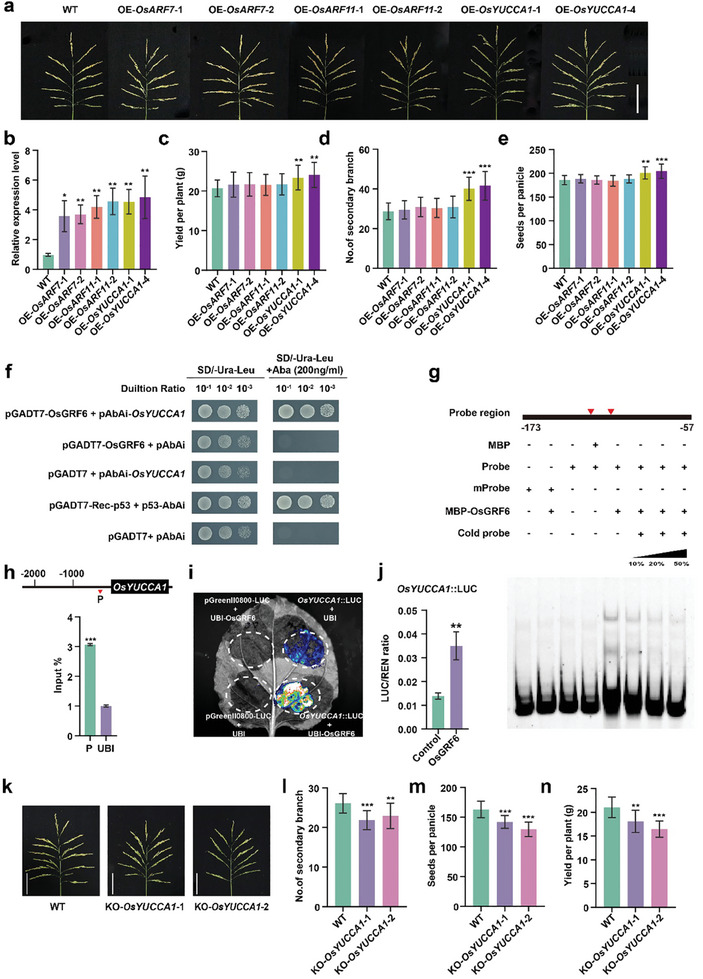
OsGRF6 targets *OsYUCCA1* to promote the secondary branch and rice grain yield. a) Phenotypes of panicles in *OsARF7*‐overexpression (OE‐*OsARF7*), *ARF11*‐overexpression (OE‐*OsARF11*) and *OsYUCCA1*‐overexpression (OE‐*OsYUCCA1*) lines. Scale bars, 10 cm. b) qRT‐PCR analysis of *OsARF7*, *OsARF11*, and *OsYUCCA1* expression in the corresponding transgenic lines. The 1 cm young inflorescences were collected for qRT‐PCR analysis. Data are mean ± s.d. (*n* = 3). c‐e) Investigation of grain yield per plant (c), seed per panicle (d), and secondary branch (e) in WT, OE‐*OsARF7*, OE‐*OsARF11*, and OE‐*OsYUCCA1* transgenic lines. Data are mean ± s.d. (*n* = 5). f) Yeast one‐hybrid analysis of OsGRF6 binding the promoter of *OsYUCCA1*. g) EMSA analysis of the interaction between OsGRF6 and the *OsYUCCA1* promoter. The mutated probe was generated by mutating all the CGC(G)A(C)G(A) motifs in the *OsYUCCA1* promoter sequence from −57 to −173 to CAC(G)A(C)G(A). h) ChIP‐qPCR validation of the OsGRF6 binding on CGC(G)A(C)G(A) sites in *OsYUCCA1* promoter. P denotes the potential target sites of *OsYUCCA1* predicted by ChIP‐qPCR analysis. The red triangle represents the binding motif “CGC(G)A(C)G(A)”. Data are mean ± s.d. (*n* = 3). i, j) Transcriptional activity of *OsYUCCA1* activated by OsGRF6 was determined in tobacco (i) and rice protoplasts (j). k) Plant phenotype comparison of WT, KO‐*OsYUCCA1*‐1, and KO‐*OsYUCCA1*‐2 lines. Scale bar, 10 cm. l‐n) Agronomic traits of WT and transgenic plants in field tests. The number of secondary branch (l), seeds per panicle (m), and grain yield per plant (n) in WT and KO‐*OsYUCCA1* mutants. Data are mean ± s.d. (*n* = 5). Different asterisks in b, c, d, e, f, h, j, l, m, and n indicate significant differences determined by Student's *t‐*test (*, *p* < 0.05; **, *p* < 0.01; ***, *p* < 0.001).

Previous studies have shown that *OsGRF6* acts as a key component in auxin biosynthesis (*OsARFs* and *OsYUCCAs*) and inflorescence architecture to control rice yield.^[^ ^39^ ^]^ To determine which genes are regulated by *OsGRF6* to influence rice yield, the relative expression levels of *OsARFs* and *OsYUCCAs* were assessed in WT, *OsGRF6*‐overexpression (OE‐*OsGRF6*) plants, and KO‐*OsGRF6* mutants using qRT‐PCR (Figure S12, Supporting Information). The *OsARF7*, *OsARF11*, and *OsYUCCA1* were the top three significantly upregulated in the OE‐*OsGRF6* line and downregulated in the KO‐*OsGRF6* mutant compared with WT (Figure S12, Supporting Information). It suggests that these three genes might be the three key direct targets of *OsGRF6* in regulating inflorescence development and rice yield. To identify which of these genes are responsible for panicle number and rice yield determination, overexpression constructs of *OsARF7*, *OsARF11*, and *OsYUCCA1* driven by the 35S promoter were individually transformed into WT plants (**Figure** **4**a,b). The grain yield of the OE‐*OsYUCCA1* transgenic lines increased by 14.7 ± 2.3% (0.01 < p < 0.001) compared with that of the WT, whereas a slight increase was observed in the OE‐*OsARF7* (4.8 ± 0.2%, P > 0.05) and OE‐*OsARF11* (4.5 ± 0.5%, P > 0.05) lines (Figure 4c). In OE‐*OsYUCCA1* transgenic lines, the numbers of secondary branches and seeds per panicle increased by 42.5 ± 3.8% (p < 0.001), and 9.1± 1.4% (0.01 < p < 0.001), respectively (Figure 4d,e), suggesting that *OsGRF6* positively regulates *OsYUCCA1* expression at the transcriptional level to control rice yield.

It is known that OsGRF6 can directly bind to CGC(G)A(C)G(A) element.^[^ ^39^ ^]^ Screening the promoter sequence revealed eight CGC(G)A(C)G(A) sites within the 2k promoter region of *OsYUCCA1* (Figure S13, Supporting Information), implying that OsGRF6 may directly bind to *OsYUCCA1*. A yeast one‐hybrid assay combined with EMSA analysis revealed that OsGRF6 could directly bind to the promoter of *OsYUCCA1* (Figure 4f,g). This was further confirmed by ChIP‐qPCR, which showed a stronger binding affinity toward the *OsYUCCA1* promoter (Figure 4h). Next, a luciferase reporter assay demonstrated that luciferase activity significantly increased upon co‐expression of UBI‐OsGRF6 with *OsYUCCA1*::LUC constructs, compared to the vector control in rice protoplasts and tobacco (Figure 4i,j). These results suggest that OsGRF6 activates the expression of *OsYUCCA1* by directly binding to the *OsYUCCA1* promoter, thereby regulating rice yield.

### 2.5 *OsYUCCA1* Promotes the Secondary Branch and Rice Grain Yield

Previous studies have shown that *OsYUCCA1* plays a crucial role in the IAA biosynthesis pathway.^[^ ^49^ ^]^ qRT‐PCR results indicated that *OsYUCCA1* is expressed at various stages of young inflorescence development, suggesting its involvement in this process (Figure S14, Supporting Information). To further understand the function of *OsYUCCA1* in controlling the number of seeds per panicle, we targeted two sites in the coding region of *OsYUCCA1* and generated two loss‐of‐function mutant alleles (KO‐*OsYUCCA1*‐1 and KO‐*OsYUCCA1*‐2) using CRISPR‐Cas9 technology (Figure S15, Supporting Information). Compared to WT, the number of secondary branches and seeds per panicle were reduced by 19.6% and 23.8% in the KO*‐OsYUCCA1*‐1 mutant, and by 28.3% and 33.8% in the KO‐*OsYUCCA1*‐2 mutant, respectively (Figure 4k–m). Consequently, the grain yield per plant of the two *OsYUCCA1* mutants decreased significantly compared to WT, with reductions of 13.4% and 15.2%, respectively (Figure 4n). These results indicate that *OsYUCCA1* positively regulates secondary branches and seeds per panicle in rice.

To explore the genetic relationship of *OsGRF6* and *OsYUCCA1*, we crossed the KO‐*OsGRF6*‐2 and OE‐*OsYUCCA1*‐1 lines (Figure S16a, Supporting Information). The KO‐*OsGRF6*‐2/OE‐*OsYUCCA1*‐1 hybrid line showed a significant increase in the number of secondary branches, seeds per panicle, and grain yield per plant compared to the WT and KO‐*OsGRF6*‐2 lines (Figure S16b–d, Supporting Information). Furthermore, the expression of *OsYUCCA1* increased in the KO‐*OsGRF6*‐2/OE‐*OsYUCCA1*‐1 line compared to the KO‐*OsGRF6*‐2 line (Figure S16e,f, Supporting Information). These results collectively demonstrate that *OsYUCCA1*, as a direct target of OsGRF6, modulates secondary branch and panicle development to enhance rice yield.

### 2.6 *OsYUCCA1* and *OsWRKY82* Synergistically Improve Rice Yield and Bacterial Blight Resistance

**Figure 5:**
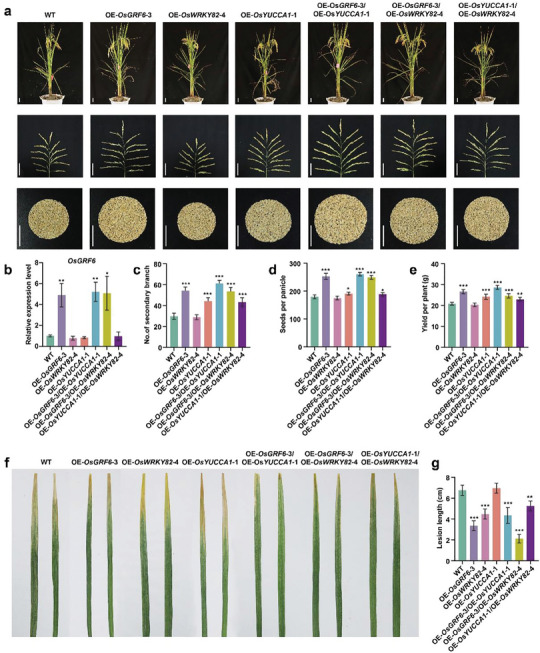
*OsGRF6* synergistically promotes both bacterial blight resistance and grain yield. a) Mature plant, panicle, and grains per plant of the WT, OE‐*OsGRF6*‐3, OE‐*OsWRKY82*‐4, OE‐*OsYUCCA1*‐1, OE‐*OsGRF6*‐3/OE‐*OsYUCCA1*‐1, OE‐*OsGRF6*‐3/OE‐*OsWRKY82*‐4 and OE‐*OsYUCCA1*‐1/OE‐*OsWRKY82*‐4 lines. Scale bar, 10 cm. b) Relative expression of *OsGRF6* in plants associated with *OsGRF6*, *OsWRKY82* and *OsYUCCA1*. The 0.5‐1 cm young inflorescences were collected for qRT‐PCR analysis. Data are mean ± s.d. (*n* = 3). c‐e) Statistical analysis of number of secondary branch (c), seeds per panicle (d) and grain yield per plant (e) in (a). Data are mean ± s.d. (*n* = 15). f, g) The phenotypes and lesion lengths of the WT, OE‐*OsGRF6*‐3, OE‐*OsWRKY82*‐4, OE‐*OsYUCCA1*‐1, OE‐*OsGRF6*‐3/OE‐*OsYUCCA1*‐1, OE‐*OsGRF6*‐3/OE‐*OsWRKY82*‐4, and OE‐*OsYUCCA1*‐1/OE‐*OsWRKY82*‐4 lines after *Xoo* PXO99 infection. The 30‐day‐old leaves were inoculated with the *Xoo* and the leaves were photographed and measured at 14 dpi. Data are mean ± s.d. (*n* = 5). Different asterisks in b, c, d, e and g indicate significant differences determined by Student's *t‐*test (*, *p* < 0.05; **, *p* < 0.01; ***, *p* < 0.001).

It has been demonstrated that OsGRF6 increases expression *OsYUCCA1* and *OsWRKY82* to increase grain yield and bacterial blight resistance. However, overexpression of *OsYUCCA1* alone did not improve bacterial blight resistance, nor did overexpression of *OsWRKY82* enhance rice yield (**Figure** **5**). To explore the genetic interactions among *OsGRF6*, *OsYUCCA1* and *OsWRKY82*—whether positive or negative—we crossed OE‐*OsGRF6*‐3, OE‐*OsYUCCA1*‐1, and OE‐*OsWRKY82*‐4 lines to generate corresponding hybrid combinations (OE‐*OsGRF6*‐3/OE‐*OsYUCCA1*‐1, OE‐*OsGRF6*‐3/OE‐*OsWRKY82*‐4 and OE‐*OsYUCCA1*‐1/OE‐*OsWRKY82*‐4) (Figure 5a). The rice yield of OE‐*OsGRF6*‐3/OE‐*OsYUCCA1*‐1 plants was higher than that of OE‐*OsGRF6*‐3 and OE‐*OsYUCCA1*‐1 lines (Figure 5c–e), accompanied by higher *OsYUCCA1* expression levels in OE‐*OsGRF6*‐3/OE‐*OsYUCCA1*‐1 plants compared to OE‐*OsYUCCA1*‐1 plants (Figure 5b; Figure S17, Supporting Information). Additionally, OE‐*OsGRF6*‐3/OE‐*OsWRKY82*‐4 plants exhibited shorter lesion lengths than OE‐*OsGRF6*‐3 or OE‐*OsWRKY82*‐4 lines upon *Xoo* inoculation (Figure 5f,g), consistent with significantly increased *OsWRKY82* expression levels in OE‐*OsGRF6*‐3 or OE‐*OsWRKY82*‐4 plants compared to OE‐*OsWRKY82*‐4 plants (Figure S18, Supporting Information). These results demonstrate that the increased resistance and yield in these hybrid lines are at least partially dependent on *OsGRF6*.

Relative to OE‐*OsWRKY82*‐4 plants, the OE‐*OsYUCCA1*‐1/OE‐*OsWRKY82*‐4 hybrids showed increased numbers of secondary branches, seeds per panicle, and grain yield per plant (Figure 5c–e). Lesion length on OE‐*OsYUCCA1*‐1/OE‐*OsWRKY82*‐4 plants were shorter than on OE‐*OsYUCCA1*‐1 plants and was intermediate between lesion length on the WT plants and the OE‐*OsGRF6*‐3 plants (Figure 5g). The expression levels of *OsGRF6*, *OsYUCCA1*, and *OsWRKY82* in OE‐*OsYUCCA1*‐1/OE‐*OsWRKY82*‐4 hybrids were similar to those in OE‐*OsYUCCA1*‐1 or OE‐*OsWRKY82*‐4 lines (Figure 5b; Figures S16 and S17, Supporting Information). These results suggest that *OsGRF6*‐mediated resistance and growth enhancement rely on *OsYUCCA1* and *OsWRKY82*, working together to synergistically improve rice yield and bacterial blight resistance.

### 2.7 Natural Variations in *OsGRF6* Gene Sequences Control Rice Resistance and Yield

**Figure 6:**
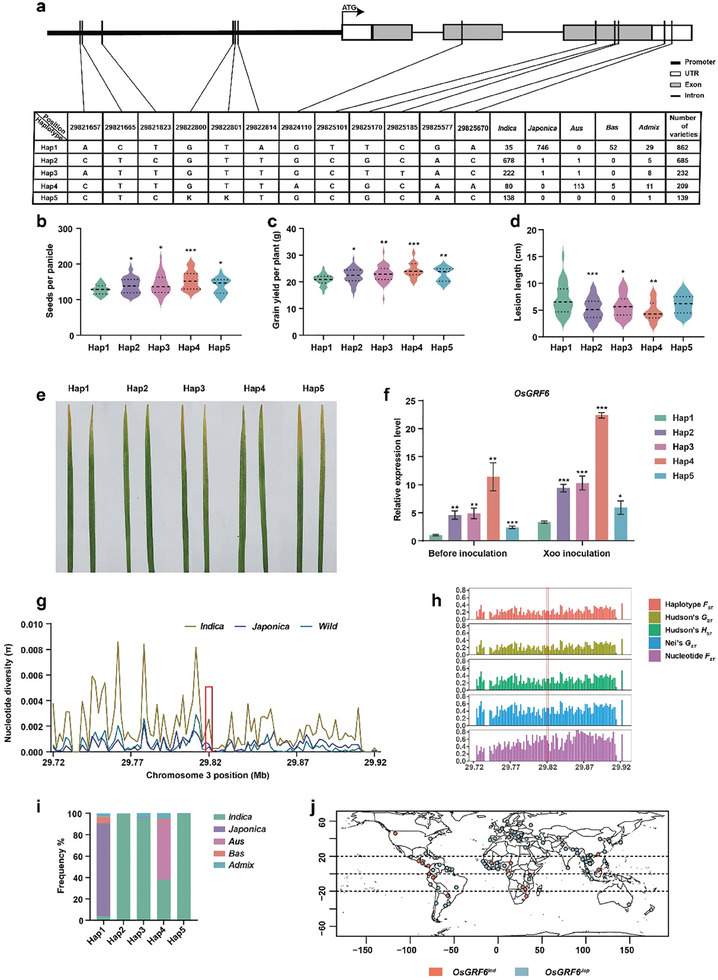
Genetic difference and association analysis of *OsGRF6*. a) Analysis of major haplotypes of the *OsGRF6* gene in 2127 cultivated accessions from the 3000 Rice Genomes Project (3K RGP). Based on 12 SNPs in *OsGRF6* upstream 2‐kb region and genomic sequences, 5 main haplotypes (frequencies > 4%) were identified. b, c) Statistical analysis of seeds per panicle (b) and grain yield per plant (c) among the five major haplotypes. n = 25 in Hap1, n = 94 in Hap2, n = 39 in Hap3, n = 14 in Hap4, n = 21 in Hap5. Data are mean ± s.d. d, e) Phenotypic comparison and lesion lengths of plants with the five major haplotypes after *Xoo* infection. The 30‐day‐old leaves were inoculated with the *Xoo* and the leaves were photographed and measured at 14 dpi. n = 25 in Hap1, n = 94 in Hap2, n = 39 in Hap3, n = 14 in Hap4, n = 21 in Hap5. Data are mean ± s.d. f) Comparison of expression levels among the major five haplotypes after *Xoo* inoculation. The 30‐day‐old leaves were inoculated with the *Xoo* for 6 days and collected for qRT‐PCR analysis. Data are mean ± s.d. (*n* = 3). g) Nucleotide diversity analysis of *OsGRF6* and its flanking region (≈200 kb). Genome sequences of 3024 cultivated germplasms and 32 wild germplasms were obtained from the Rice SNP‐Seek Database (https://snpseek.irri.org/_snp.zul) and OryzaGenome (http://viewer.shigen.info/oryzagenome/). h) Genetic differentiation parameters between *indica* and *japonica* ecotypes for *OsGRF6* and its flanking genomic regions. The red box indicates the position of *OsGRF6*. i) Distribution ratio of the five haplotypes (*indica, japonica, Aus*, *Bas*, *Admix*) among the 2127 accessions. j) Geographical distribution of *OsGRF6^Ind^* and *OsGRF6^Jap^* haplotypes. The types of *OsGRF6^XI^* and *OsGRF6^GJ^* were indicated by orange and cyan box, respectively. Different asterisks in b, c, d, and f indicate significant differences determined by Student's *t‐*test (*, *p* < 0.05; **, *p* < 0.01; ***, *p* < 0.001).

Based on *OsGRF6*’s pivotal role in mediating secondary branching and bacterial blight resistance in rice, we aimed to explore the elite haplotype of *OsGRF6* for rice resistance and yield based on genetic difference across *Oryza sativa*. According to the upstream 2‐kb region and genomic sequences of this gene in 3024 rice accessions from the 3000 Rice Genomes Project (3K RGP), 12 SNPs were identified and categorizing them into 5 main haplotypes (Hap1‐Hap5) with frequencies > 4% (**Figure** **6**a). Expression analysis revealed that *OsGRF6* ^Hap4^ exhibited the highest expression activity, while *OsGRF6* ^Hap1^ showed the weakest (Figure S19, Supporting Information). Accessions with different *OsGRF6* haplotypes displayed significant variation in seeds per panicle and grain yield per plant (Figure 6b,c). Additionally, we observed an increase in rice resistance from *OsGRF6* ^Hap1^ to *OsGRF6* ^Hap4^ (Figure 6d,e), with *OsGRF6* ^Hap4^ accessions displaying the shortest lesion lengths after *Xoo* inoculation and the most pronounced change in *OsGRF6* expression (Figure 6d–f), Similarly, the downstream target gene *OsWRKY82* is also significantly induced in *OsGRF6* ^Hap4^ (Figure S20, Supporting Information). These findings collectively indicate that *OsGRF6* ^Hap4^ enhances both rice resistance and yield through increased transcriptional activity.

To investigate the genetic difference of *OsGRF6* in rice germplasm, we analyzed the 200 kb region of this gene in 3024 cultivated accessions, which included cultivars from five different groups. We noted an imbalance in the distribution of two major types (*indica* and *japonica*) of *OsGRF6* across the five haplotypes (Figure 6g–i). The *indica* type predominantly harbored four haplotypes (Hap2‐Hap5) of *OsGRF6* (96.96%) (Figure 6i; Figure S21, Supporting Information), while the *japonica* type was predominantly associated with Hap1 (99.73%) (Figure 6i; Figure S21, Supporting Information) suggesting significant subspecies differentiation of the *OsGRF6* gene. To validate this hypothesis, we analyzed sequence variations within this gene and its flanking regions across the 3024 cultivated rice varieties. The nucleotide diversity value (π) of *OsGRF6* in *japonica* cultivated rice populations was markedly lower than that in *indica* populations (Figure 6g; Table S1, Supporting Information). Moreover, genetic differentiation parameters of the *OsGRF6* locus between *indica* and *japonica* subspecies, including haplotype and nucleotide *F_ST_*, Nei's *G_ST_* and Hudson's *G_ST_* and *H_ST_*, indicated substantial differentiation (Figure 6h; Table S2, Supporting Information). Genetic analysis showed that the five parameters in the *OsGRF6* locus were all greater than 0.25 between *indica* and *japonica* subspecies (Figure 6h; Table S2, Supporting Information), with haplotype ‐based and nucleotide ‐based *F_ST_* values notably reaching 0.518 and 0.703, respectively(Figure 6h; Table S2, Supporting Information), indicating significant genetic differentiation among populations^[^ ^50^ ^]^ (Figure 6h; Table S2, Supporting Information). Furthermore, we observed a trend of increasing *OsGRF6^Jap^* proportion with latitude (Figure 6j), indicating regional differentiation in *OsGRF6* distribution. These results underscore the pronounced genetic differentiation at the *OsGRF6* locus between *indica* and *japonica* rice subspecies, paving the way for the development of new germplasm with improved yield and disease resistance by leveraging its superior haplotype.

## 3 Discussion

Worldwide rice production faces increasing challenges from various diseases, necessitating the urgent development of disease‐resistant crops for sustainable agriculture. Despite the discovery of numerous genes associated with disease resistance over recent decades, understanding the genes and molecular mechanisms that synergistically enhance both rice yield and disease resistance remains limited. In this study, we found that *OsGRF6* as a novel gene that plays a synergistic role in improving both yield and bacterial blight resistance in rice. Multiple lines of evidence indicate that *OsGRF6* regulates secondary branch development and bacterial blight resistance by controlling the expression of downstream genes *OsYUCCA1* and *OsWRKY82*, respectively (Figures 2 and 4). Notably, upon pathogen attack, the expression of *OsGRF6* is significantly induced, leading to a rapid activation of *OsWRKY82* expression and thereby enhancing bacterial blight resistance (Figure 5). In contrast, the expression level of *OsYUCCA1* remains relatively stable before and after pathogen infection (Figure 5), indicating that *OsGRF6* primarily promotes normal plant development under normal growth conditions. However, once pathogen infection, *OsGRF6* becomes crucially activated, contributing to both bacterial blight resistance and maintaining rice development under diverse environmental conditions.

Plant hormones, also known as phytohormones, are signaling compounds that regulate crucial aspects of growth, development and biotic/abiotic stress responses.^[^ ^51^, ^52^ ^]^ *OsGRF6* promotes secondary branch development and increases rice yield by regulating auxin biosynthesis.^[^ ^39^ ^]^ In this study, we identified *OsYUCCA1*, as a key target gene of OsGRF6 that regulates grain yield (Figure 4). *OsYUCCA1* encodes a flavin monooxygenase‐like enzyme involved in IAA biosynthesis via a tryptophan‐dependent pathway.^[^ ^49^ ^]^ Consistent with the phenotypes of *OsGRF6* and *OsYUCCA1* transgenic lines (Figure 1l–o, 4a–e,k–n), the IAA content was significantly increased in OE‐*OsGRF6* and OE‐*OsYUCCA1* compared to the wild‐type (Figure S22, Supporting Information), while IAA content was reduced in both KO‐*OsGRF6* and KO‐*OsYUCCA1* (Figure S22, Supporting Information). Transcriptome analysis between the OE‐*OsYUCCA1* and WT plants showed that *OsYUCCA1* influences other genes related to auxin synthesis and metabolism (Figure S23a, Supporting Information). Seedlings of the *OsYUCCA1* transgenic line treated with different concentrations of IAA showed that *OsYUCCA1* expression was affected by IAA (Figure S23b‐d, Supporting Information), indicating that the *OsGRF6*‐*OsYUCCA1* module modulates rice yield by influencing auxin synthesis. In plant immunity, jasmonic acid (JA) is recognized as a key regulator in defense responses to pathogens.^[^ ^53^, ^54^ ^]^ The expression of *OsWRKY82* was enhanced by JA treatment,^[^ ^46^ ^]^ suggesting the involvement of the *OsGRF6*‐*OsWRKY82* module in the JA signaling pathway for rice bacterial blight resistance. Similar to *OsWRKY82*, *OsGRF6* is significantly induced after JA treatment (Figure S24a, Supporting Information), and the lesion lengths in *OsGRF6* and *OsWRKY82* transgenic plants were significantly shortened after exogenous JA treatment following bacterial infection (Figure S24b,c, Supporting Information). A Combined transcriptome analysis identified four genes (*AOS2*, *AOS3*, *LOX6*, and *LOX9*) related to JA synthesis or metabolism that are common targets of OsGRF6 and OsWRKY82 (Figure S24d, Supporting Information). qRT‐PCR results showed that these four genes are regulated by *OsWRKY82*, with varying expression levels in *OsWRKY82* transgenic plants, especially after *Xoo* treatment (Figure S24e–h, Supporting Information). Further research is needed to determine if these genes are directly targeted by *OsWRKY82* to regulate rice bacterial blight resistance.

Rice grain yield is largely determined by the capacity of the sink, the strength of the source, and the flow of photo‐assimilates.^[^ ^55^ ^]^ Source strength is influenced by the capacity of roots and leaves to capture and assimilate nutrients, while sink capacity is a product of effective panicle number, grain number per panicle, and grain size or weight.^[^ ^56^ ^]^ Highly productive rice typically has a strong source with high nitrogen utilization and/or photosynthetic efficiency, as well as a large sink with increased grain number and/or weight,^[^ ^57^ ^]^ just as shown as the *HPY1*, which can synergistically increase photosynthetic rate and seed size to increase rice yield.^[^ ^58^ ^]^ In this study, photosynthetic efficiency analysis at the seedling stage showed significant improvement in the *OsGRF6* overexpression line compared to WT (Figure S25a, Supporting Information). Similar trends were observed at the heading stage (Figure S25b, Supporting Information). Transcriptome data analysis at the seedling stage revealed that many differentially expressed genes (DEGs) are involved in various photosynthesis processes (Figure S25c, Supporting Information). These results suggest that *OsGRF6* might enhance source strength by increasing photosynthetic efficiency, thereby boosting sink capacity and improving rice yield. However, as a nuclear‐localized transcription factor, *OsGRF6* lacks a chloroplast localization signal. How *OsGRF6* enhances photosynthetic efficiency through nucleocytoplasmic shuttling is a scientific question worth exploring.

*OsGRF6* is targeted by miR396b to promote inflorescence branching and increase rice yield.^[^ ^39^ ^]^ In our study, after *Xoo* treatment, the miR396b target mimicry of miR396b (MIM396) lines showed shorter lesion lengths and lower bacterial growth rates compared to WT (Figure S26a–c, Supporting Information). Along with improved bacterial blight resistance, ROS accumulation increased, and ROS scavenging enzyme activity significantly decreased in MIM396 plants (Figure S26d–f, Supporting Information). qRT‐PCR analysis demonstrated that *OsGRF6* and *OsWRKY82* were upregulated in MIM396 lines, especially under *Xoo* treatment (Figure S26g,h, Supporting Information), indicating that miR396b is involved in the *OsGRF6*‐*OsWRKY82* module to coordinate yield and resistance.

**Figure 7:**
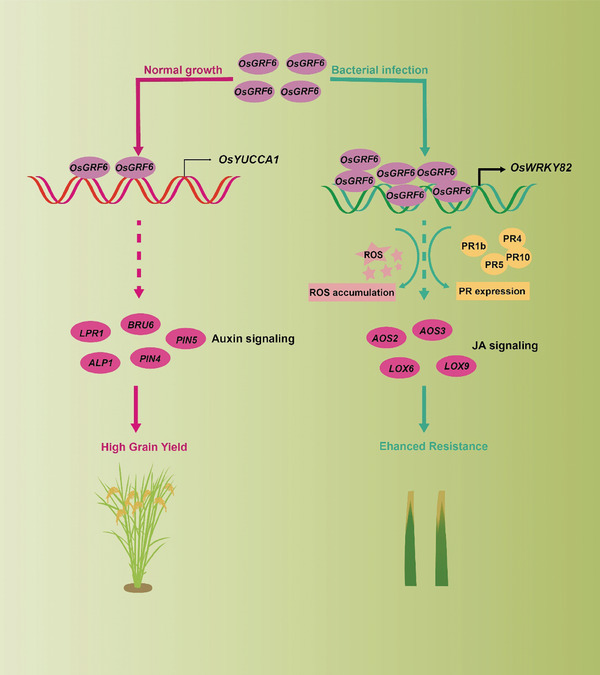
Proposed working model for *OsGRF6* enhancing grain yield and bacterial blight resistance in rice. Under normal circumstances, *OsGRF6* regulates the expression of its downstream target gene *OsYUCCA1*, which in turn modulates genes involved in auxin metabolism, promoting the development of rice secondary branches to enhance grain yield. However, upon pathogen infection, while the expression level of *OsYUCCA1* remains relatively constant, *OsGRF6* transcription significantly increases. This upregulation activates *OsWRKY82* expression, triggering the activation of disease‐responsive genes and accumulation of H_2_O_2_. Subsequently, *OsGRF6* regulates genes in the JA metabolic pathway, thereby participating in the resistance process in rice.

Achieving coordinated improvement of rice yield and resistance is crucial for sustainable rice cultivation and the balanced development of food security and population sustainability. GRF family genes play vital roles in plant growth, development, and environmental stress responses.^[^ ^29^, ^30^, ^33^ ^]^ In rice, *OsGRF4*, *OsGRF6* and *OsGRF8* increase grain size and weight, elongate panicles and promote inflorescence branching to enhance yield.^[^ ^35^, ^38^, ^39^ ^]^ Additionally, OsGRF8 directly targets the *F3H* gene to mediate resistance to brown planthopper.^[^ ^59^ ^]^ Here, we found that *OsGRF6*, as a resistance gene, regulates the expression of auxin and JA signaling pathway genes by targeting downstream *OsYUCCA1* and *OsWRKY82*, thereby synergistically enhancing both rice yield and bacterial blight resistance. (**Figure** **7**). Co‐overexpression of *OsYUCCA1* and *OsWRKY82* resulted in higher yields and stronger resistance than the overexpression of either gene alone (Figure 7; Figures S27 and S28, Supporting Information). Moreover, the OE‐*OsYUCCA1*‐1/OE‐*OsWRKY82*‐4 lines did not completely restore the yield and resistance phenotypes of the OE‐*OsGRF6*‐3 line (Figure 5), indicating that *OsGRF6* may also regulate yield and resistance through other minor effect genes (*OsARF7*, *OsARF11*, and *LOC_Os04g57760*) in addition to the primary effect genes *OsYUCCA1* and *OsWRKY82*. Haplotype analysis revealed that the *OsGRF6* ^Hap4^ haplotype shows distinct growth advantages with high yield and bacterial blight resistance (Figure 6a–f). Furthermore, *OsGRF6* comprises the *OsGRF6^Ind^* and *OsGRF6^Jap^* subtypes, exhibiting distinct regional distribution differentiation (Figure 6g–j). This implies that *OsGRF6* has significant breeding potential if we effectively exploring the elite haplotype in rice population.

## 4 Conclusion

In summary, our results elucidate the mechanistic framework by which the *OsGRF6*‐*OsYUCCA1*/*OsWRKY82* module regulates rice yield and bacterial blight resistance. *OsGRF6* enhances rice yield and resistance by targeting downstream genes *OsYUCCA1* and *OsWRKY82*, respectively. This study reveals a novel mechanism through which the high‐yield *OsGRF6* gene regulates bacterial blight resistance in rice. These findings deepen our understanding of the genetic mechanisms governing rice yield and disease resistance and provide new theoretical support for the genetic improvement of rice.

## 5 Experimental Section

### Plant Material and Growth Conditions

The rice (*Oryza sativa*) varieties used in this work included Yuetai B (YB), the target mimicry of miR396b (MIM396) lines,^[^ ^39^ ^]^ overexpression lines (OE‐*OsGRF6*, OE‐*OsYUCCA1*, and OE‐*OsWRKY82*) and CRISPR‒Cas9‐edited mutants (KO‐*OsGRF6*, KO‐*OsYUCCA1*, and KO‐*OsWRKY82*) were grown in the experimental field of Wuhan University in Lingshui (Hainan Province) and Wuhan (Hubei Province). For pathogen infection, seeds were germinated and seedlings were grown in a growth chamber at 28 °C for 30 days (14‐h light/10‐h dark), maintaining a relative humidity of 60%.

### Plasmid Construction

The promoters of *OsYUCCA1* and *OsWRKY82* were first amplified from YB genomic DNA and ligated into pGreenII0800‐LUC and pAbAi vectors, respectively, to generate *OsYUCCA1*::LUC, *OsWRKY82*::LUC, pAbAi‐*OsYUCCA1*, and pAbAi‐*OsWRKY82*. The full‐length coding sequences (CDSs) of *OsYUCCA1* and *OsWRKY82* were amplified from YB cDNA templates using Phanta Max Super‐Fidelity DNA Polymerase (Vazyme, Nanjing, China). *OsYUCCA1* and *OsWRKY82* were then inserted into pCAMBIA1301‐UBI and HBT‐sGFP vectors, respectively, while *OsWRKY82* was also ligated into pGBKT7 and pGADT7 vectors. gRNA constructs required for CRISPR/Cas9‐mediated generation of *OsYUCCA1* and *OsWRKY82* mutant alleles were designed as Ma et al. described.^[^ ^60^ ^]^

### Pathogen Inoculation

To evaluate rice bacterial blight disease resistance, 30‐day‐old seeding plants were inoculated with the *Xoo* strain PXO99 using the leaf‐clipping method.^[^ ^61^ ^]^ The strain was cultured on potato‐based medium for 2 days at 28 °C, then suspended in sterilized water to an OD_600_ of 0.8. The bacterial growth rate in rice leaves was photographed and measured at 14 days post‐inoculation (dpi).

To measure bacterial growth, the inoculated leaves were thoroughly ground using a mortar and pestle, and the ground tissue was suspended in 1 mL of ddH_2_O. Then, the suspensions were then diluted and transferred to potato‐based medium plates. The plates were incubated at 28 °C in the dark for 3 days and colonies were counted.

### RNA Isolation and Quantitative Real‐Time PCR (qRT–PCR) Analysis

Total RNAs were extracted from rice tissues using TRIzol Reagent (Invitrogen, Carlsbad, CA, USA) following to manufacturer's protocol. Complementary cDNA was synthesized with 1 µg RNA using HiScript III First Strand cDNA Synthesis Kit (+gDNA Wiper) (Vazyme, Nanjing, China). qRT–PCR was performed using the HieffqPCR SYBR Green Master Mix (No Rox) (Yeasen, Shanghai, China) and the Roche LightCyclerr 480II instrument. The rice *ubiquitin* gene was used as the internal control and the relative expression levels were calculated using the 2^−ΔΔCT^ method.

### RNA‐Sequencing Analysis

The inoculated leaves and 1 cm young inflorescence s were used for total RNA‐sequencing. A total of 3 µg RNA was used for construction of the RNA‐sequencing with three replicates. Then, RNA‐sequencing were performed by igenecode company (Beijing, China). The libraries were sequenced using DNBSEQ‐T7 platform. The filtered clean reads were mapped to the *Nipponbare* genome sequence (http://rice.uga.edu/index.shtml). Differentially expressed genes were identified using a threshold of (|log2 (FoldChange)| > 1 and q‐value < 0.001). The FPKM value was used to analysis the transcript abundance, with each analysis based on three independent biological replicates.

### Endogenous Substance Measurement

To measure endogenous substances, 30‐day‐old rice seedlings inoculated with *Xoo* for 6 days were used. The inoculated leaves were collected and subjected to measurement of H_2_O_2_ content, and analysis of catalase (CAT) and peroxidase (POD) activity. The endogenous substance contents were measured using absorbance methods with spectrophotometry, employing the corresponding assay kits from Beijing Boxbio Science Technology (Beijing, China).

### Subcellular Localization

For subcellular localization in rice protoplast, the protoplasts were prepared and transfected with HBT‐OsYUCCA1 and HBT‐OsWRKY82 fusion vectors by PEG method, then cultured at 28 °C overnight. The GFP fluorescence was observed using the FV1000 confocal system (Leica, Germany).

### Yeast One‐Hybrid Assay

The yeast one‐hybrid assays were conducted according to the protocol specified in the Matchmaker Gold Yeast One‐Hybrid Library Screening System User Manual (Clontech). The full‐length coding sequence of *OsGRF6* was cloned into pGADT7. First, the yeast strain Y1HGold was transformed with the recombinant vectors pAbAi‐*OsYUCCA1* and pAbAi‐*OsWRKY82* to determine the minimum inhibitory concentration of AbA. Then, pAbAi‐*OsYUCCA1* was co‐transformed with AD, pAbAi‐*OsWRKY82* with AD, and pAbAi with AD‐OsGRF6 into the yeast strain Y1HGold to assess their self‐activation properties. Subsequently, the pAbAi‐*OsYUCCA1* and pAbAi‐*OsWRKY82* constructs were introduced into the yeast strain Y1HGold (Weidi Biotechnology, Shanghai, China) together with pGADT7‐OsGRF6. Colonies that grew on synthetic defined (SD) medium lacking Ura and Leu were then transferred to SD/‐Ura‐Leu selection medium containing Aureobasidin A (AbA; Coolaber, Beijing, China) to confirm DNA–protein interactions.

### Dual‐Luciferase Transcriptional Activity Assay

The transcriptional activity of *OsYUCCA1* and *OsWRKY82* was assessed as described previously.^[^ ^62^ ^]^ To analyze their activity in rice protoplasts, OE‐*OsGRF6* vectors were constructed as effectors. *OsYUCCA1*::LUC and *OsWRKY82*::LUC constructs were co‐transfected into rice protoplasts with OE‐*OsGRF6* and cultured overnight at 28 °C. The LUC/REN activities were measured using the dual‐luciferase reporter assay system (Promega, Madison, WI, USA).

For detection of transactivation in tobacco (*Nicotiana benthamiana*), recombinant plasmids were transformed into GV3101 (pSoup‐p19) chemically competent cells (Weidi Biotechnology, Shanghai, China) and infiltrated into tobacco leaves. LUC signals were detected 3 days post‐infiltration using the Tanon 5200 Multi imaging system.

### Electrophoretic Mobility Shift Assay (EMSA)

The full‐length of *OsGRF6* was cloned into the pMAL‐c2X vector with a MBP tag. The recombinant MBP‐OsGRF6 protein was expressed in the *Escherichia coli* BL21 (DE3) and purified according to the manufacturer's instructions. FAM‐labeled primers, including both forward and reverse strands, were synthesized by GenScript (Nanjing, China), with an unlabeled probe serving as a competitor. For the DNA EMSA, the purified recombinant protein was incubated with FAM‐labelled probes in a binding buffe at 25 °C for 30 min. The mixture was then resolved using 5% native PAGE electrophoresis in 0.5× TBE buffer for 1 h. Visualization was performed using the Typhoon Trio Imager (GE, Amersham Typhoon).

### ChIP–qPCR Analysis

Chromatin immunoprecipitation coupled with quantitative polymerase chain reaction (ChIP‐qPCR) was performed as performed as described previously with minor modifications.^[^ ^42^ ^]^ Approximately 2 g of seedling leaves and 1 cm young inflorescence s of OE‐*OsGRF6* transgenic plants were used. In brief, plant materials were crosslinked with 1% (v/v) formaldehyde (Sigma, 47 608) under vacuum for 20 min, followed by random shearing of the genomic DNA to 100–400 bp through sonication. The samples were incubated with anti‐Flag antibodies (MBL, PM020) for precipitation and purification of the protein/chromatin complexes. The enriched DNA products were then used for qRT‐PCR analysis.

### Transcriptional Activation Analysis

To assess the transcriptional activation of *OsWRKY82* in rice protoplasts, the CDS region of *OsWRKY82* was fused with the GAL4 DNA‐binding domain and the GAL4BD‐VP16 domain. These recombinant plasmids were then transferred into rice protoplasts. Relative LUC activity was determined by calculating the ratio of LUC/REN using the dual‐luciferase reporter assay system (Promega, Madison, WI, USA).

### Haplotype and Evolutionary Analysis

For *OsGRF6*‐based association analysis, a 2 kb promoter region and the genomic sequences of 3024 rice accessions were obtained from the RFGB v2.0 database.^[^ ^63^ ^]^ A total of 12 single nucleotide polymorphisms (SNPs) were identified within the *OsGRF6* gene region, spanning from the 5′ promoter region to the 3′ end of the genomic sequences. For haplotype analysis, the 2127 accessions were divided into five haplotypes based on these 12 SNPs. The seeds per panicle, grain yield per plant, and lesion length of the 193 accessions were determined among these haplotypes.

The genomic sequences of 3056 rice accessions (3024 cultivated rice varieties and 32 wild rice varieties) were subjected to genetic diversity analysis. Nucleotide diversity (π) in the 200‐kb region spanning *OsGRF6* in wild rice *XI*, and *GJ* subspecies were calculated with 2‐kb sliding window and 2‐kb step. Genetic differentiation parameters (haplotype and nucleotide *F_ST_*, Nei's *G_ST_*, and Hudson's *G_ST_* and *H_ST_*) between *XI*, and *GJ* varieties in the OsGRF6 region and its flanking regions were analyzed using the PopGenome R software package.^[^ ^64^ ^]^

### Statistical Analysis

All assays were conducted with at least three replicates per line. GraphPad Prism 9 software was used to calculate the means (± SD) from three independent experiments. Statistical significance was determined using Student's *t*‐test, with significance levels defined as follows: *, *p* < 0.05; **, *p* < 0.01 and ***, *p* < 0.001. The primers used for transgenic assay, qRT‐PCR assay, subcellular localization, luciferase activity assay ChIP‐qPCR, EMSA and yeast transformation were listed in Table S3 (Supporting Information).

## Conflict of Interest

The authors declare no conflict of interest.

## Author Contributions

H.Y., M.C., and F.F. contributed equally to this work (co‐first authors). S.L. and H.Y. designed the experiments. F.F., M.C., and H.Y. performed the experiments. M.C., and X.Z. carried out the bioinformatic analyses. H.Y., F.F., and R.W. analyzed the data. F.S., X.L., N.L., and X.Z. work on transgenic lines and participated in other molecular experiments. H.Y. and S.L. wrote the paper. H.Y., M.C., F.F., and S.L. revised the paper. All authors discussed and commented on the manuscript.

## Supporting information



Supporting Information

## Data Availability

The data that support the findings of this study are available from the corresponding author upon reasonable request.
