# Supplementary material for: OsGRF6‐OsYUCCA1/OsWRKY82 Signaling Cascade Upgrade Grain Yield and Bacterial Blight Resistance in Rice
Source: Adv Sci (Weinh). 2024 Oct 23;11(46):2407733. doi: 10.1002/advs.202407733 (PMC11633520; doi:10.1002/advs.202407733)
Supplement: Supplementary file 1 — Supporting Information [file ADVS-11-2407733-s001.docx]

**Supporting Information**


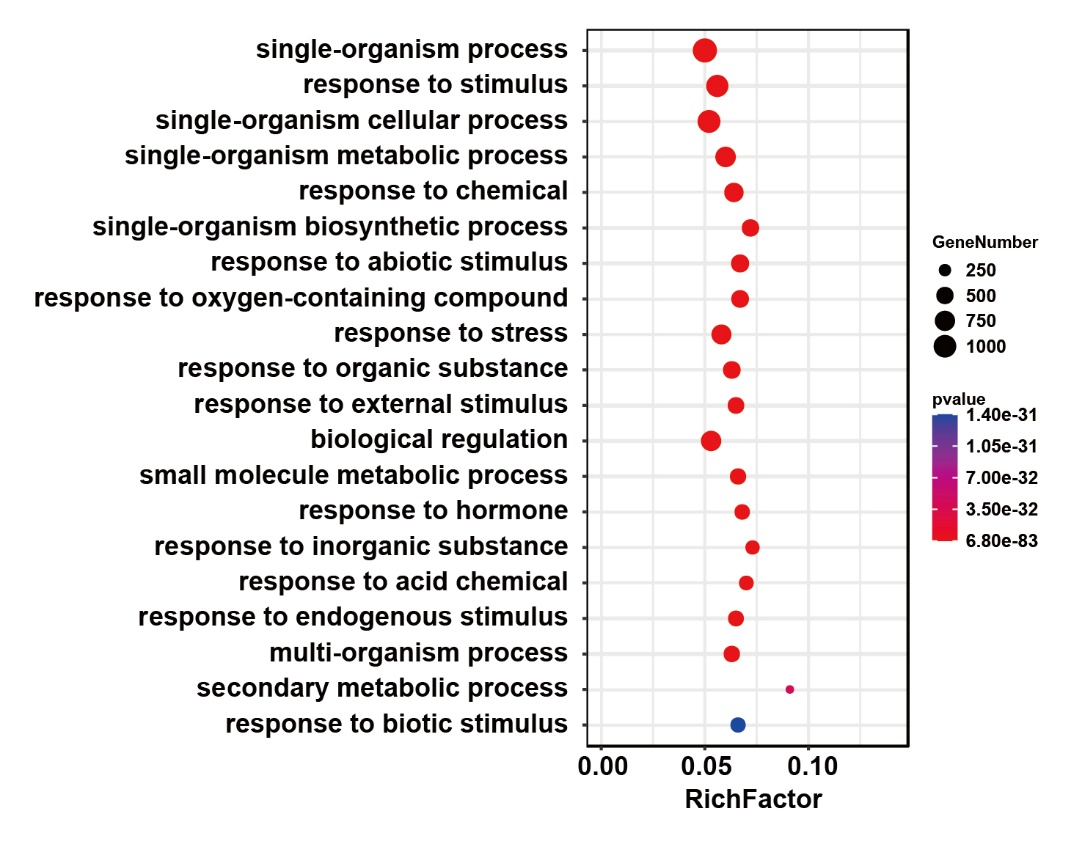


Figure S1. GO term analysis of *OsGRF6*-regulated genes after *Xoo* infection. The 30-day-old seedling leaves were collected for RNA-sequencing and GO analysis.


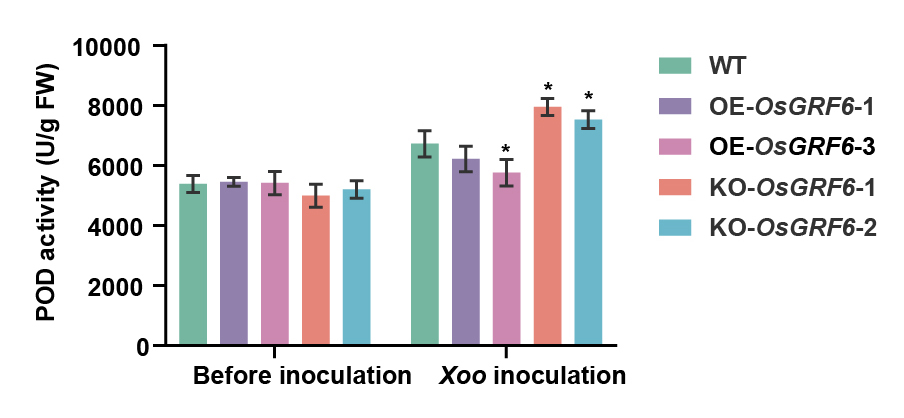


Figure S2. Measurement of Peroxidase (POD) activity in *OsGRF6* transgenic lines. Data are mean ± s.d. (*n* = 3). Different asterisks indicate significant differences determined by Student’s *t-*test (*, *P* < 0.05).


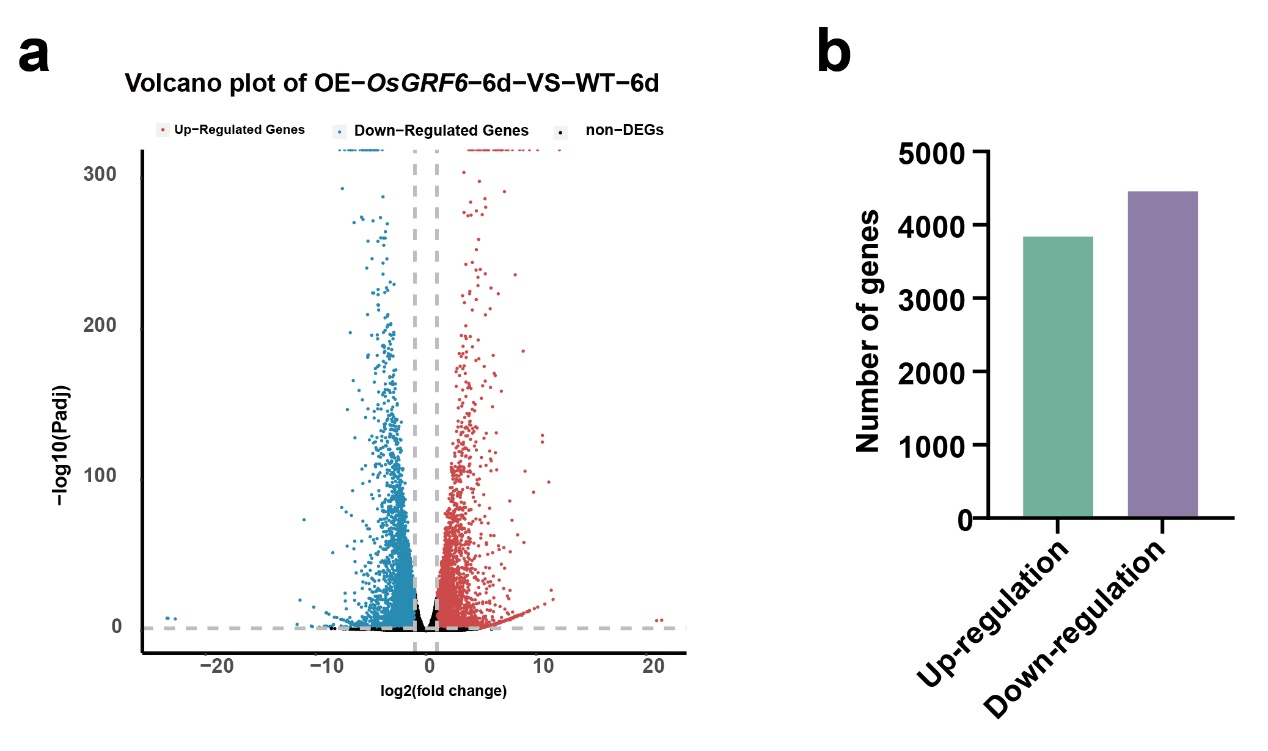


Figure S3. Transcriptome analysis of (WT, YB) VS OE-*OsGRF6* after *Xoo* infection for 6 days. a) Volcano plot of differentially expressed genes (DEGs). b) Statistical diagram of the number of DEGs. The 30-day-old leaves were inoculated with the *Xoo* and collected for transcriptome analysis.


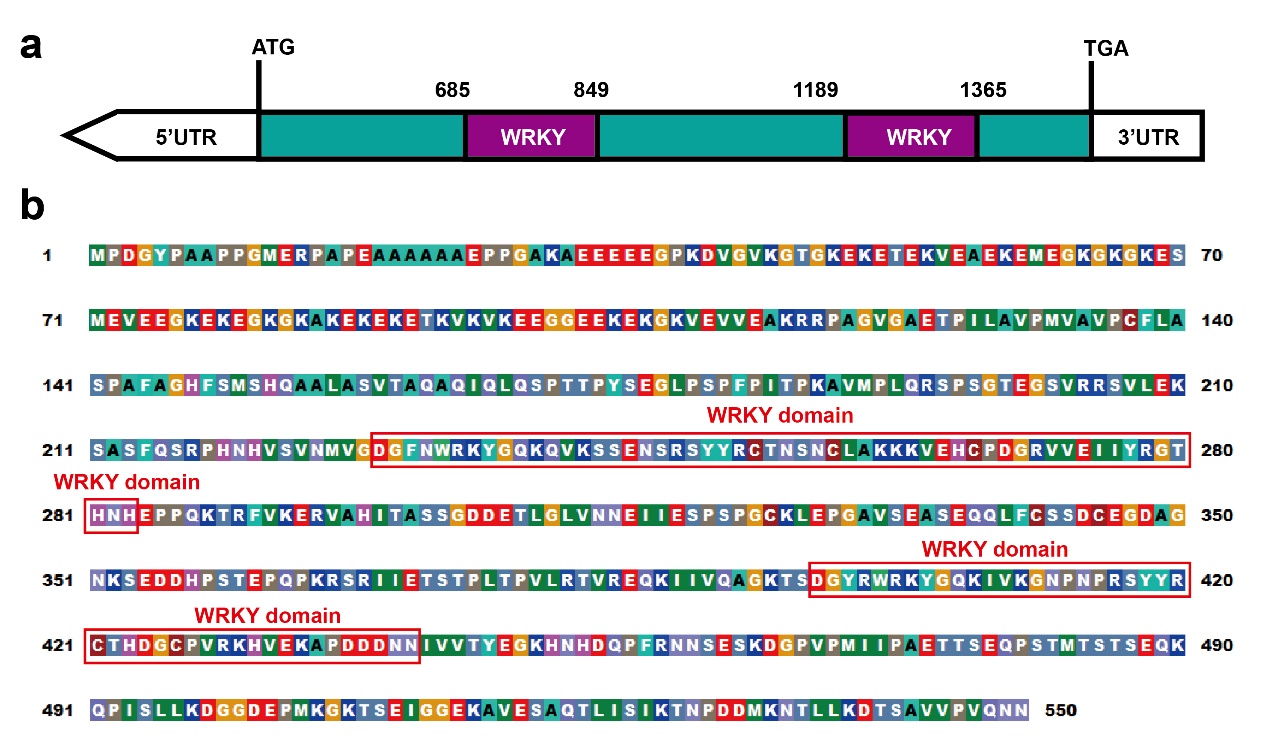


Figure S4. Analysis of gene structure of *OsWRKY82*. a) The gene structure of *OsWRKY82*. b) Analysis of the WRKY domain in OsWRKY82 protein sequence. The red box indicates the WRKY domain.


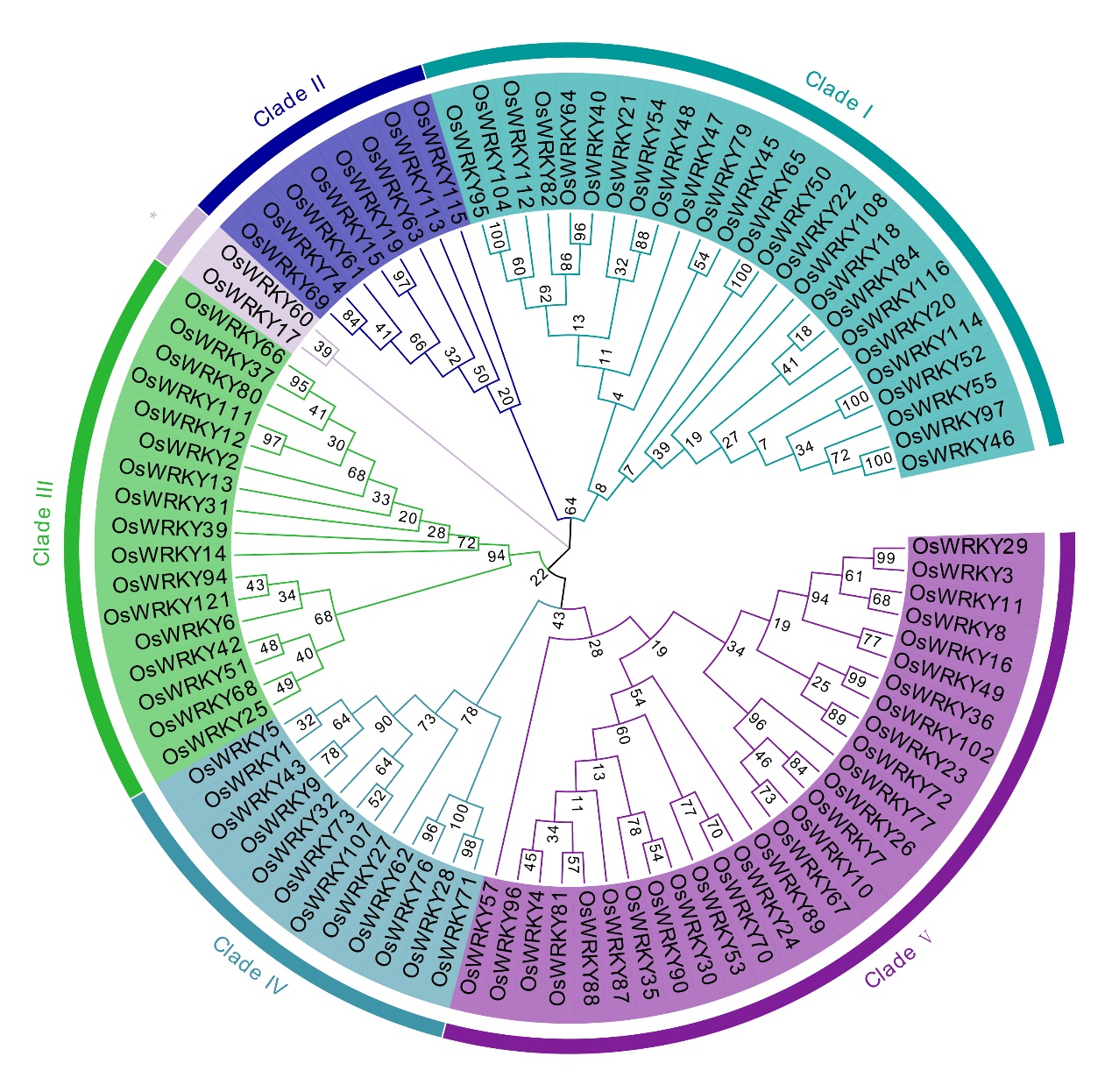


Figure S5. Phylogenetic analysis of the OsWRKY82 homologous protein in rice. Multiple sequence alignment of the *Nipponbare* genome sequences was performed using ClustalW. A Maximum Likelihood phylogenetic tree was constructed with MEGA 7.0, using default parameters and 1,000 bootstrap replicates. The tree is based on the amino acid sequences of the WRKY conserved domain, with the numbers representing bootstrap support from 1,000 replicates.


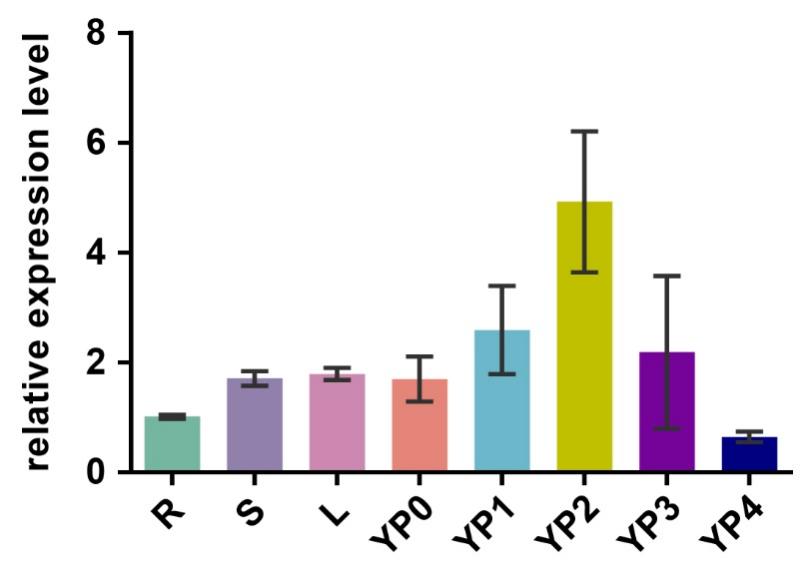


Figure S6. qRT–PCR analysis of the *OsWRKY82* expression in various organs. R, roots; C, clums; L, leaves; YP0, YP1, YP2, YP3 and YP4 represent young inflorescences about 0 - 0.5 cm, 0.5 - 1 cm, 1 - 2 cm, 2 – 3 cm and 3 – 4 cm respectively. Data are mean ± s.d. (*n* = 3).


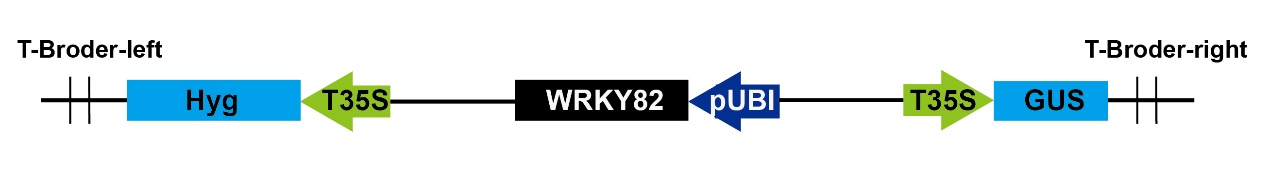
Figure S7. Model structure of *OsWRKY82* gene in overexpressed rice materials.


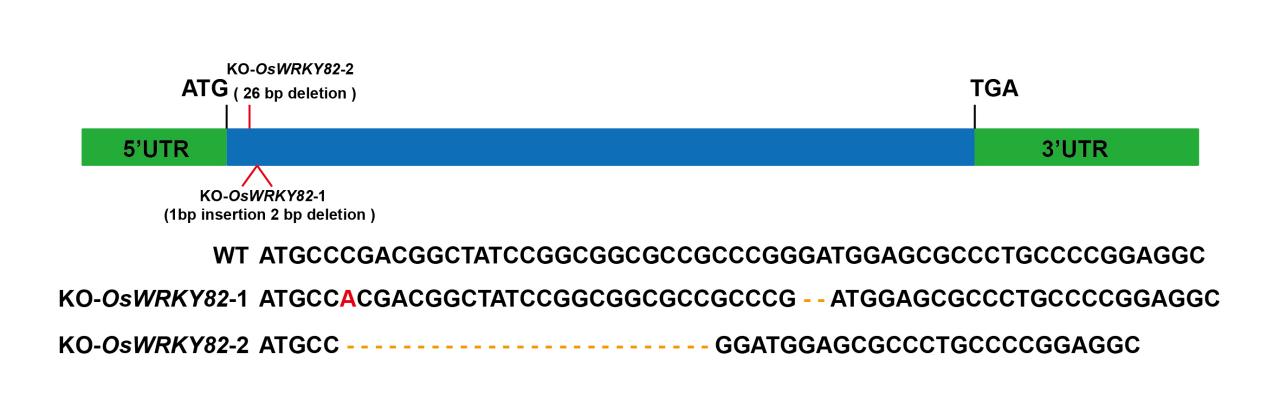


Figure S8. Diagram of the CRISPR/Cas9-mediated targeted mutagenesis of *OsWRKY82*. Upper: schematic diagram indicating the *OsWRKY82* gene harboring the CRISPR/Cas9 target sites. Lower: the alignment between WT and mutated (KO-*OsWRKY82*-1 and KO-*OsWRKY82*-2) sequences containing the target sites.


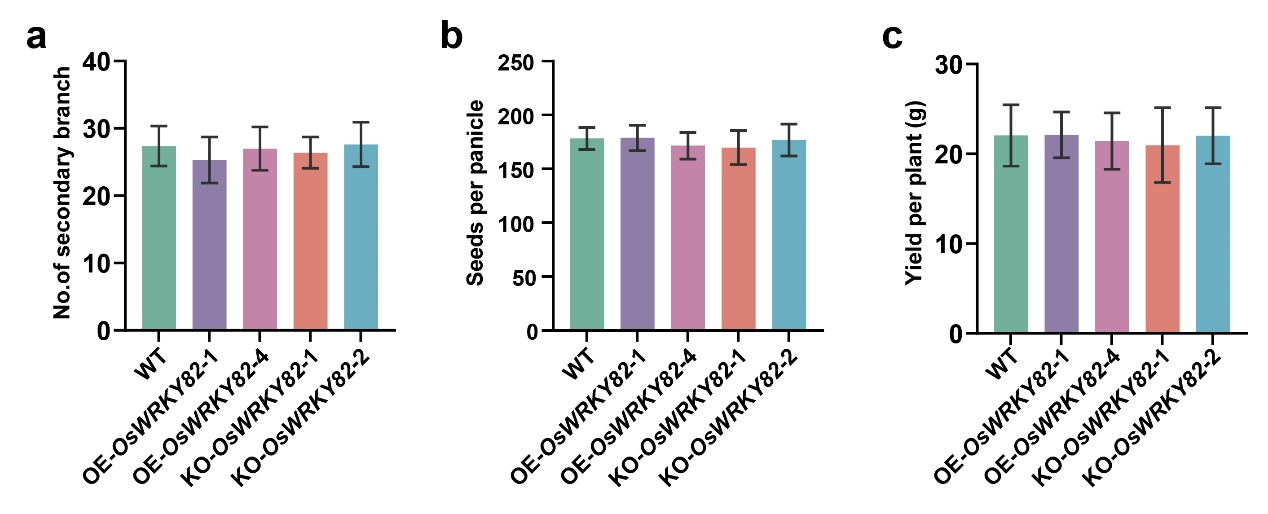


Figure S9. The phenotype of the WT and *OsWRKY82* transgenic lines. Statistical analysis of number of secondary branch (a), seeds per panicle (b) and grain yield per plant (c) in *OsWRKY82* transgenic lines. Data are mean ± s.d. (*n* = 15).


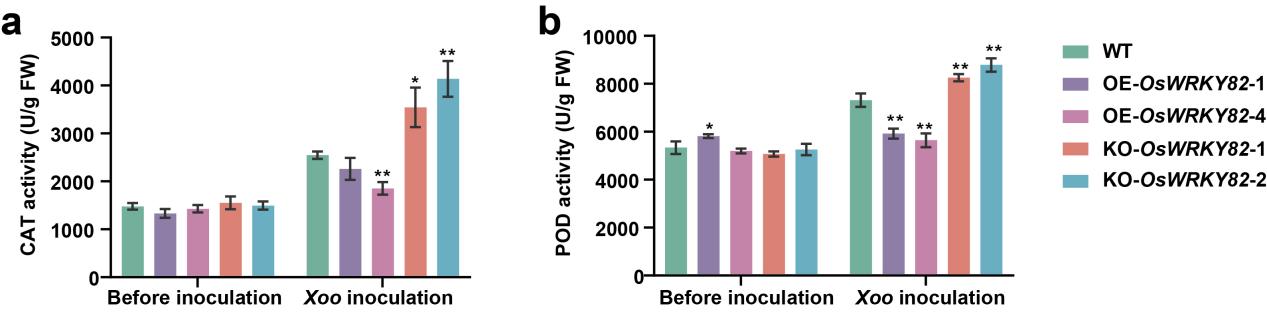


Figure S10. Analysis of ROS-scavenging enzyme activity in *OsWRKY82* transgenic lines. a, b) Measurement of CAT (a) and POD (b) in 30-d seedlings (WT, OE-*OsWRKY82* and KO-*OsWRKY82*) after *Xoo* infection for 6 days. Data are mean ± s.d. (*n* = 3). Asterisks indicate significant differences determined by Student’s *t*-test (*, *P* < 0.05; **, *P* < 0.01).


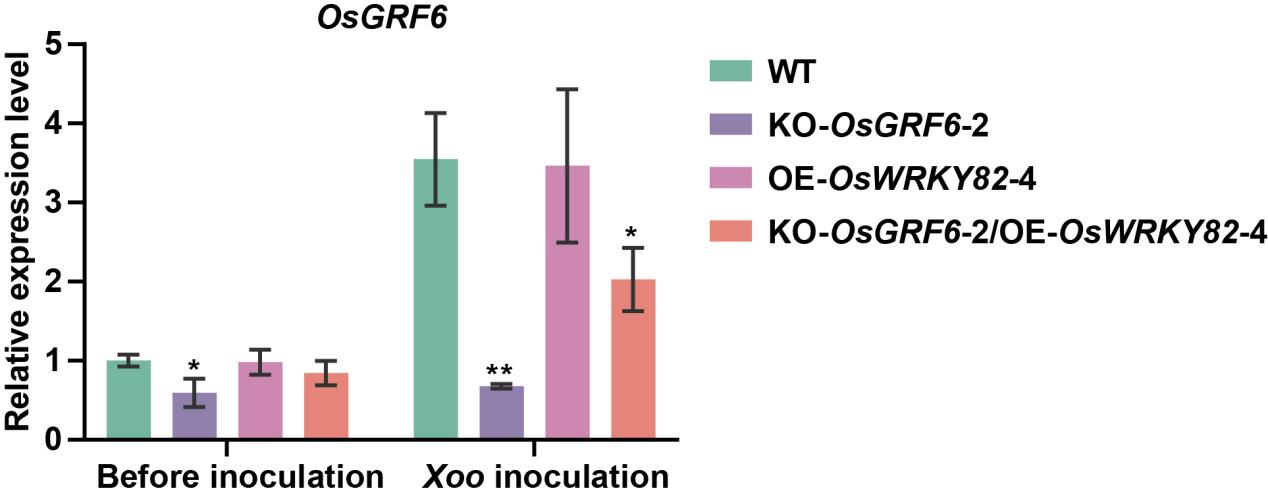


Figure S11. qRT-PCR analysis of *OsGRF6* expression in the WT, KO-*OsGRF6*-2, OE-*OsWRKY82*-4 and KO-*OsGRF6*-2/ OE-*OsWRKY82*-4 lines after *Xoo* inoculation. The 30-day-old leaves were inoculated with the *Xoo* for 6 days and collected for qRT-PCR analysis. Data are mean ± s.d. (*n* = 3). Different asterisks indicate significant differences determined by Student’s *t-*test (*, *P* < 0.05; **, *P* < 0.01).


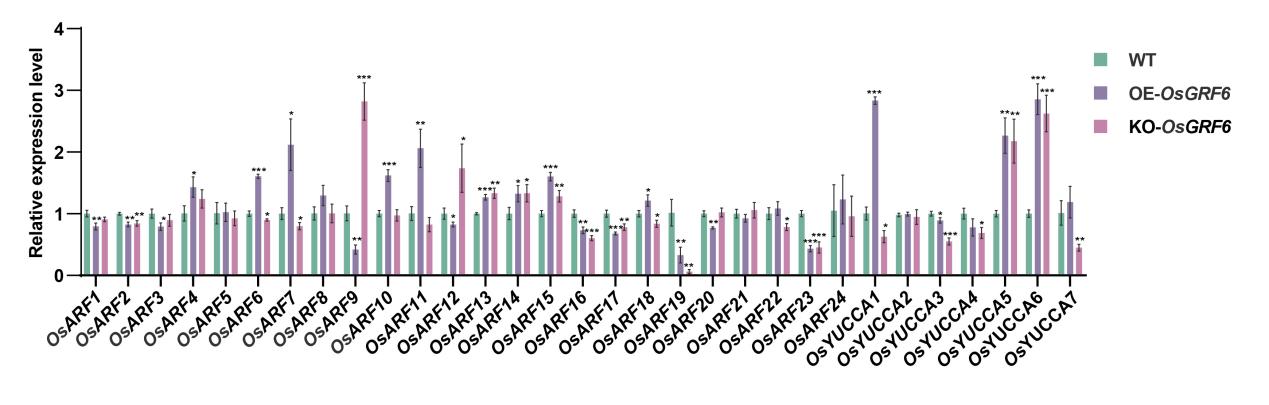


Figure S12. Relative expression of *OsARFs* and *OsYUCCAs* in OE-*OsGRF6* transgenic lines and KO-*OsGRF6* mutant. 1 cm young inflorescence were collected for qRT-PCR analysis. Data are mean ± s.d. (*n* = 3). Different asterisks indicate significant differences determined by Student’s *t-*test (*, *P* < 0.05; **, *P* < 0.01; ***, *P* < 0.001)


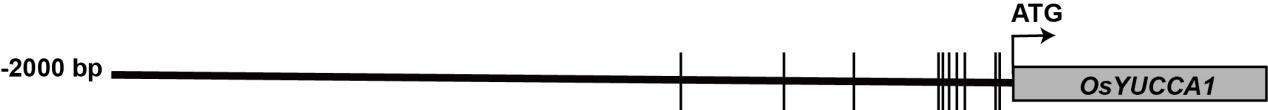


Figure S13. Bioinformatic analysis of the CGC(G)A(C)G(A) motif on the *OsYUCCA1* promoter. The lines represent the positions of the CGC(G)A(C)G(A) motif.


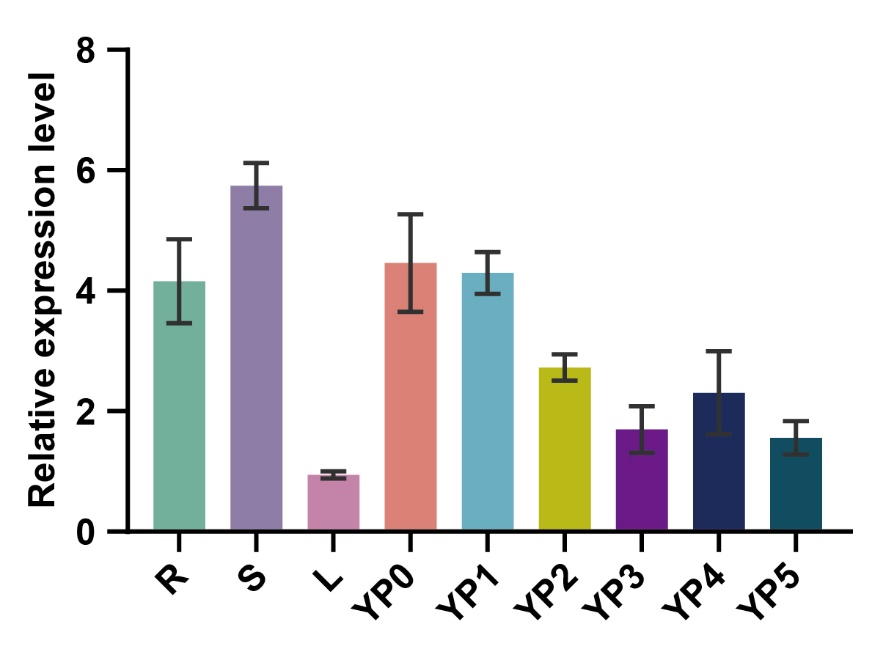


Figure S14. qRT–PCR analysis of the *OsYUCCA1* expression in various organs. R, roots; C, clums; L, leaves; YP0, YP1, YP2, YP3, YP4 and YP5 represent young inflorescences about 0 - 0.5 cm, 0.5 - 1 cm, 1 - 2 cm, 2 – 3 cm, 3 – 4 cm and 4 - 5 cm respectively. 1 cm young inflorescences were collected for qRT-PCR analysis. Data are mean ± s.d. (*n* = 3).


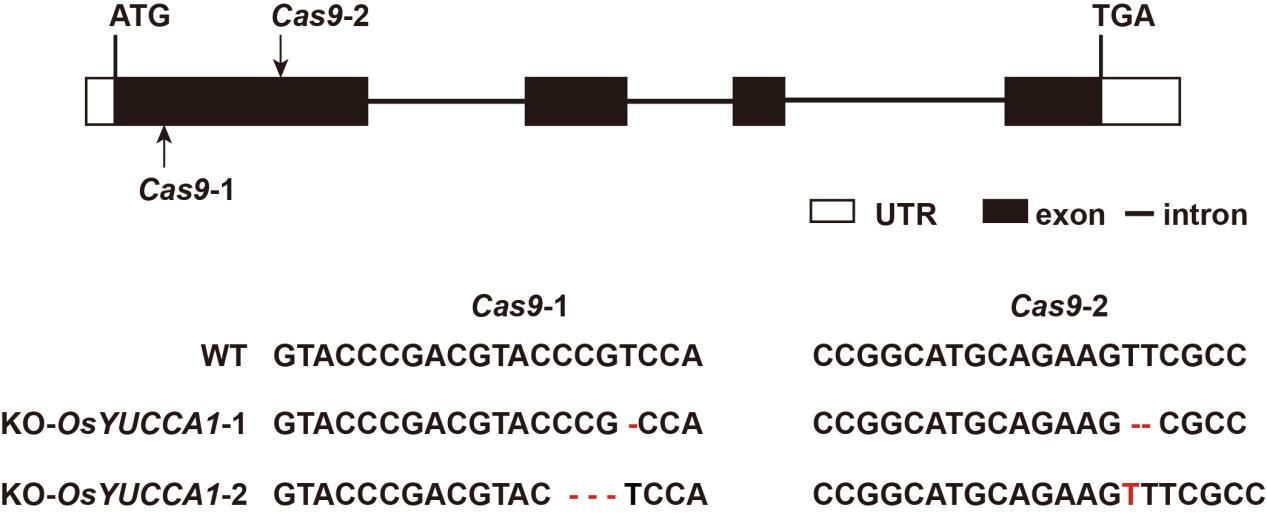


Figure S15. Diagram showing CRISPR-Cas9-mediated mutations in KO-*OsYUCCA1*-1 and KO-*OsYUCCA1*-2 lines. The arrow indicated the CRISPR/Cas9 target sites. Exons, introns, and UTRs are represented by black boxes, lines, and white boxes, respectively.


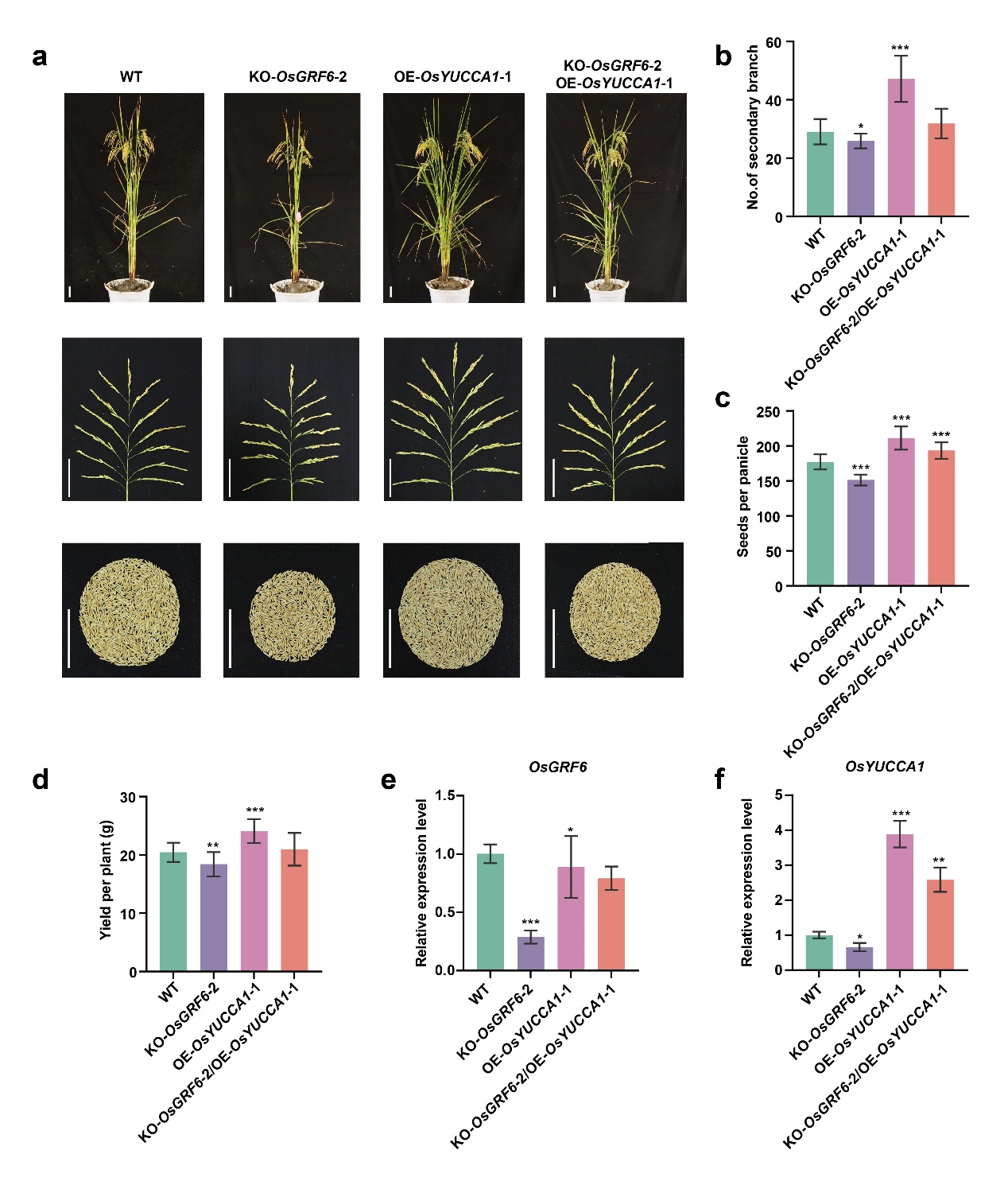


Figure S16. Analysis the genetic relationship between *OsGRF6* and *OsYUCCA1*. a) Plant phenotypes and grain yield of WT, KO-*OsGRF6*-2, OE-*OsYUCCA1*-1 and KO-*OsGRF6*-2/OE-*OsYUCCA1*-1 lines. Scale bar, 10 cm. b-d) Statistical analysis of number of secondary branch (b), seeds per panicle (c) and grain yield per plant (d) in WT, KO-*OsGRF6*-2, OE-*OsYUCCA1*-1 and KO-*OsGRF6*-2/OE-*OsYUCCA1*-1 plants. Data are mean ± s.d. (*n* = 15). e, f) Expression level of *OsGRF6* (e) and *OsYUCCA1* (f) in the WT, KO-*OsGRF6*-2, OE-*OsYUCCA1*-1 and KO-*OsGRF6*-2/OE-*OsYUCCA1*-1 lines. 1 cm young inflorescences were collected for qRT-PCR analysis. Data are mean ± s.d. (*n* = 3). Different asterisks indicate significant differences determined by Student’s *t-*test (*, *P* < 0.05; **, *P* < 0.01; ***, *P* < 0.001).


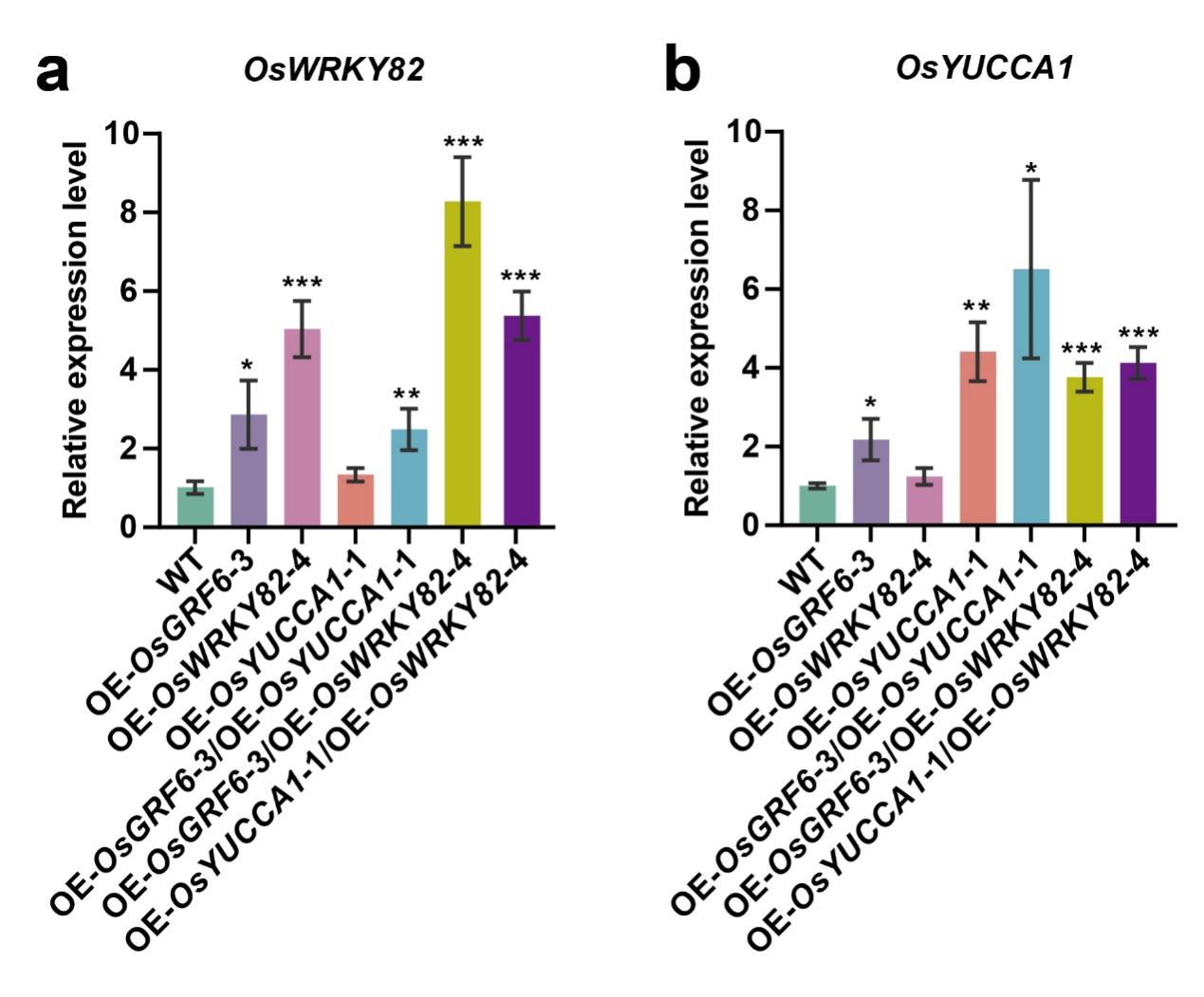


Figure S17. The relative expression of *OsWRKY82* and *OsYUCCA1* in *OsGRF6, OsWRKY82* and *OsYUCCA1* associated plants with young inflorescences (0.5–1 cm). a, b) qRT–PCR analysis of the *OsWRKY82* (a) and *OsYUCCA1* (b) expression level in young inflorescences (0.5–1 cm). Data are mean ± s.d. (*n* = 3). Different asterisks indicate significant differences determined by Student’s *t-*test (*, *P* < 0.05; **, *P* < 0.01; ***, *P* < 0.001).


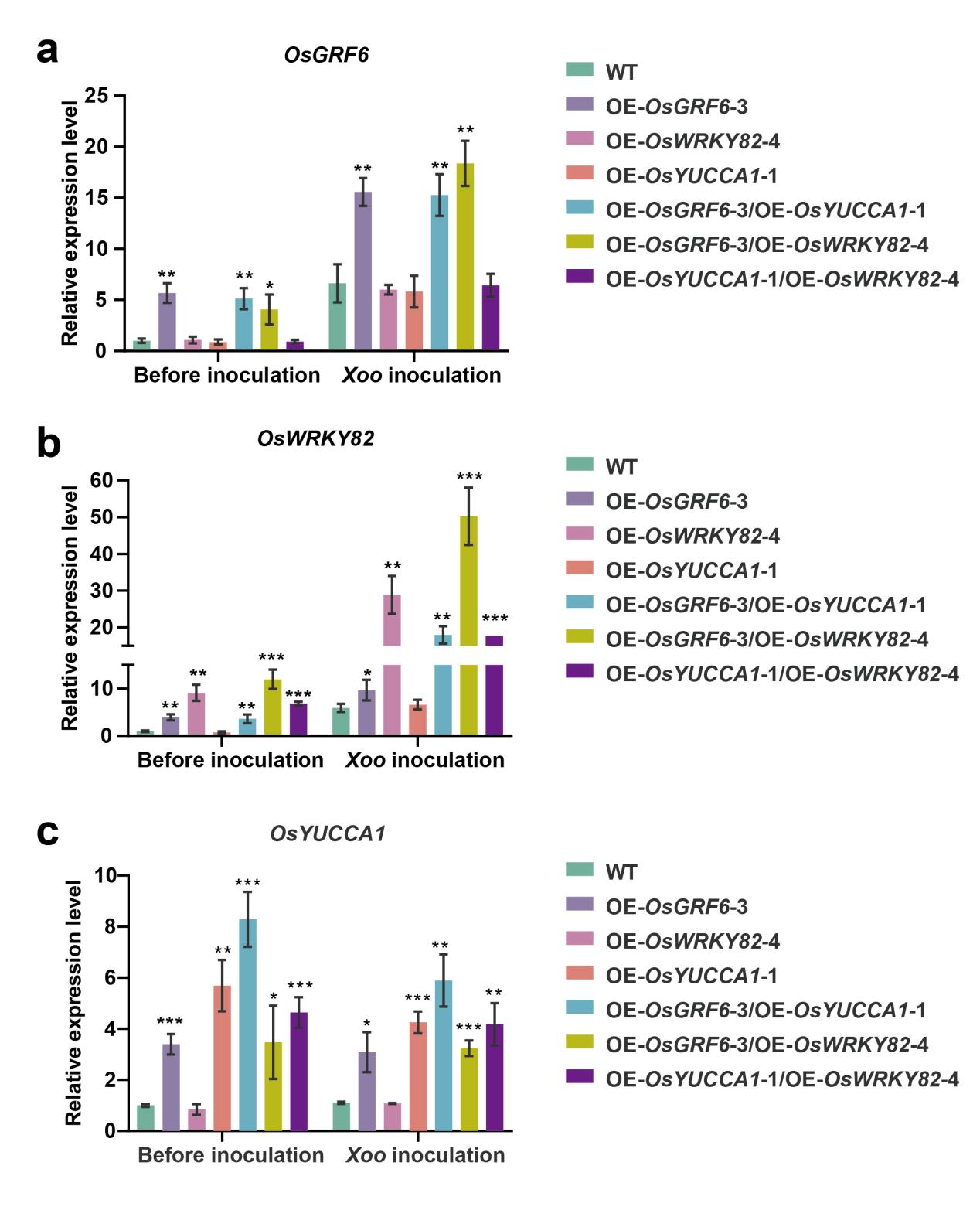


Figure S18. *OsGRF6*, *OsWRKY82* and *OsYUCCA1* expression levels in WT, OE-*OsGRF6*-3, OE-*OsWRKY82*-4, OE-*OsYUCCA1*-1, OE-*OsGRF6*-3/OE-*OsYUCCA1*-1, OE-*OsGRF6*-3/OE-*OsWRKY82*-4 and OE-*OsYUCCA1*-1/OE-*OsWRKY82*-4 lines after *Xoo* infection. a-c) Expression level of *OsGRF6* (a), *OsWRKY82* (b) and *OsYUCCA1* (c) in in plants associated with *OsGRF6*, *OsWRKY82* and *OsYUCCA1* associated plants on the 6th day after *Xoo* infection. Different asterisks indicate significant differences determined by Student’s *t-*test (*, *P* < 0.05; **, *P* < 0.01; ***, *P* < 0.001). Data are mean ± s.d. (*n* = 3).


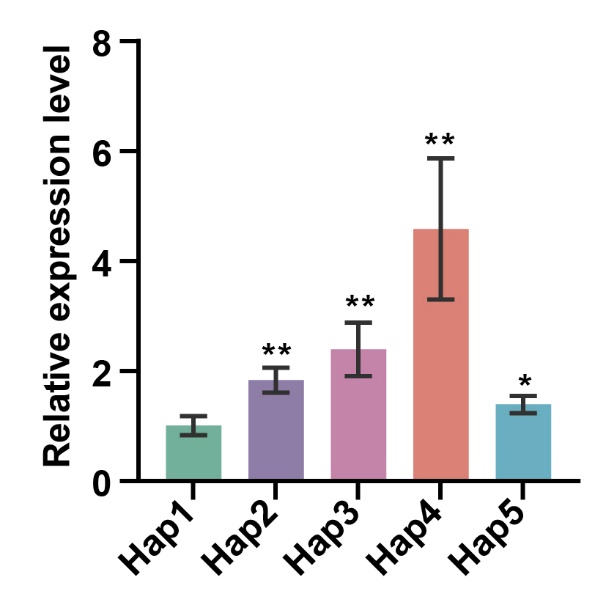


Figure S19. Relative expression level of *OsGRF6* in the major five haplotypes. The expression level was performed with young inflorescences (0.5–1 cm) of field-grown rice plants at the booting stage. Different asterisks indicate significant differences determined by Student’s *t-*test (*, *P* < 0.05; **, *P* < 0.01). Data are mean ± s.d. (*n* = 3).


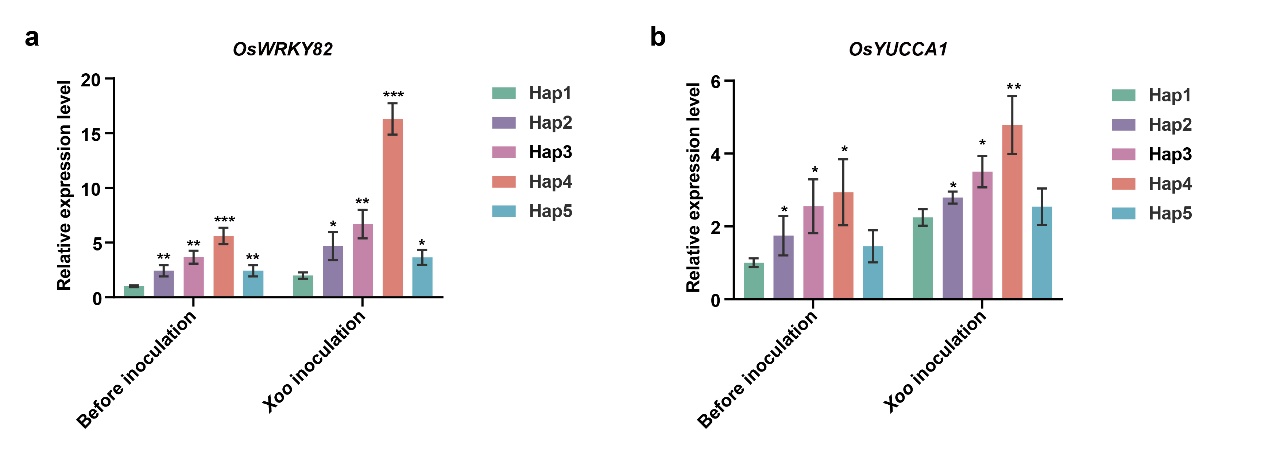


Figure S20. Comparison of *OsWRKY82* and *OsYUCCA1* expression levels among the major five haplotypes after *Xoo* inoculation. Expression level of *OsWRKY82* (a) and *OsYUCCA1* (b) were measured in the associated plants on the 6th day post-*Xoo* infection. Data are mean ± s.d. (*n* = 3). Different asterisks indicate significant differences determined by Student’s *t-*test (*, *P* < 0.05; **, *P* < 0.01; ***, *P* < 0.001).


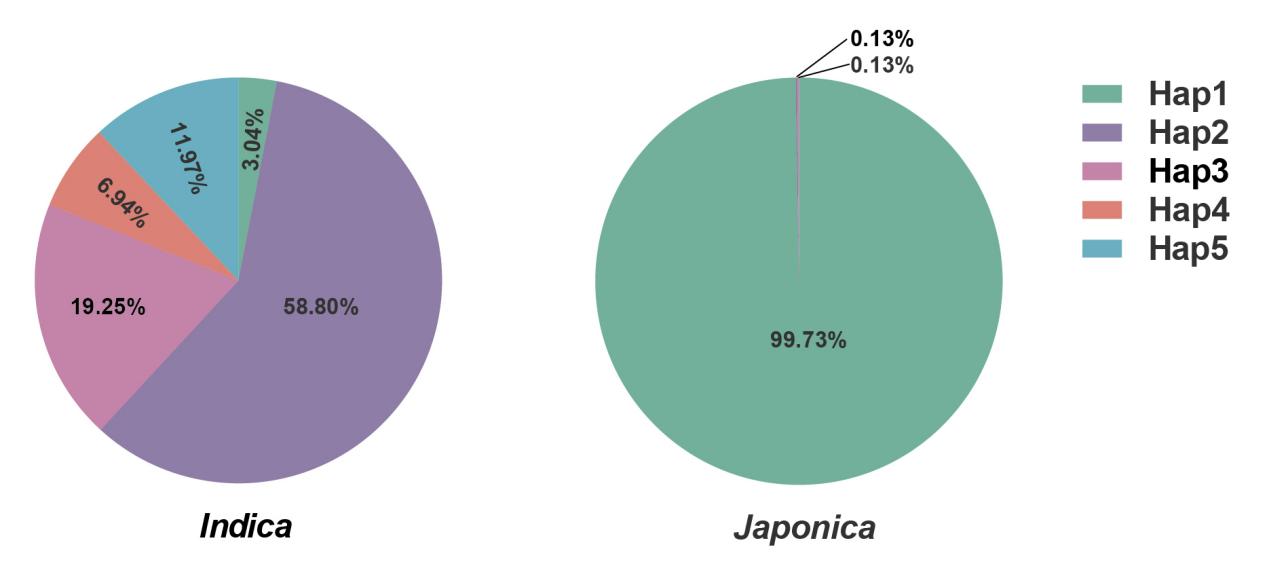


Figure S21. Distribution frequency of the five haplotypes in *indica* and *japonica* population.


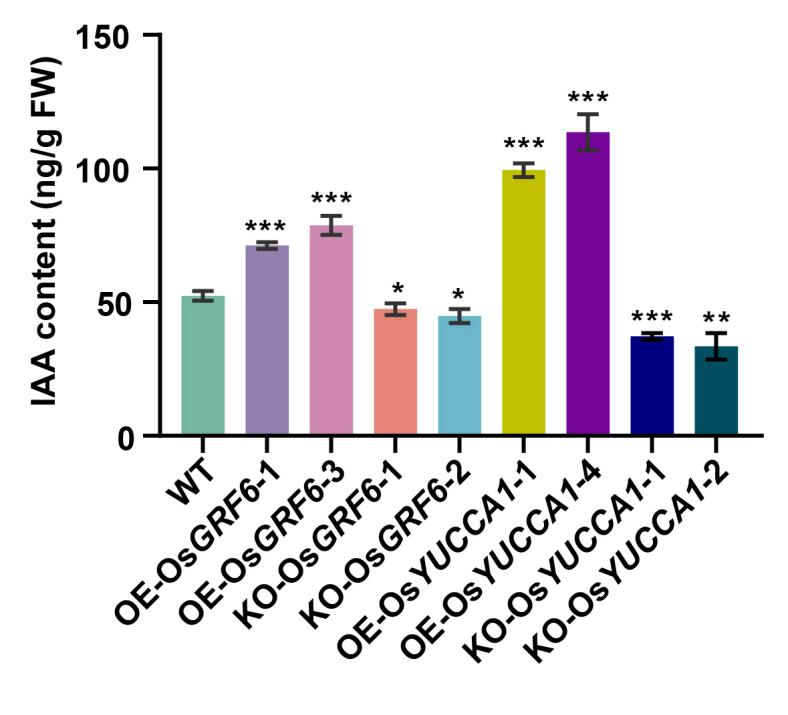


Figure S22. Content of IAA in young inflorescences (0.5–1 cm) of WT, *OsGRF6* and *OsYUCCA1* transgenic lines. The young inflorescences (0.5–1 cm) were collected for measurement of IAA content. Data are mean ± s.d. (*n* = 3). Different asterisks indicate significant differences determined by Student’s *t-*test (*, *P* < 0.05; **, *P* < 0.01; ***, *P* < 0.001).


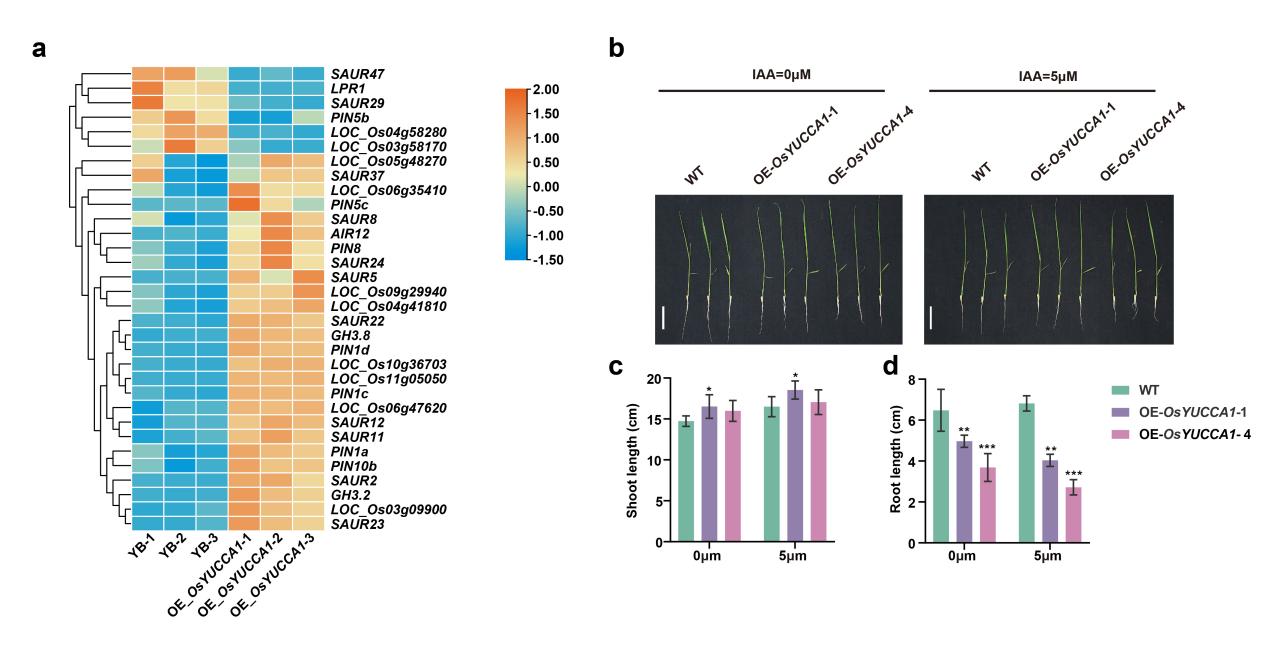


Figure S23. *OsYUCCA1* is involved in auxin signaling pathway. a) Heatmaps of DEGs clustering to auxin signaling pathway in YB VS OE-*OsYUCCA1*. b) Characterization of root length in WT and OE-*OsYUCCA1* lines under control, 5 mM IAA treatments for 7 days. Scale bars, 5 cm. c, d) Statistical analysis of shoot length (c) and root length (d) in WT and OE-*OsYUCCA1* lines under IAA treatment for 7 days. Different asterisks indicate significant differences determined by Student’s *t-*test (*, *P* < 0.05; **, *P* < 0.01; ***, *P* < 0.001). Data are mean ± s.d. (*n* = 3).


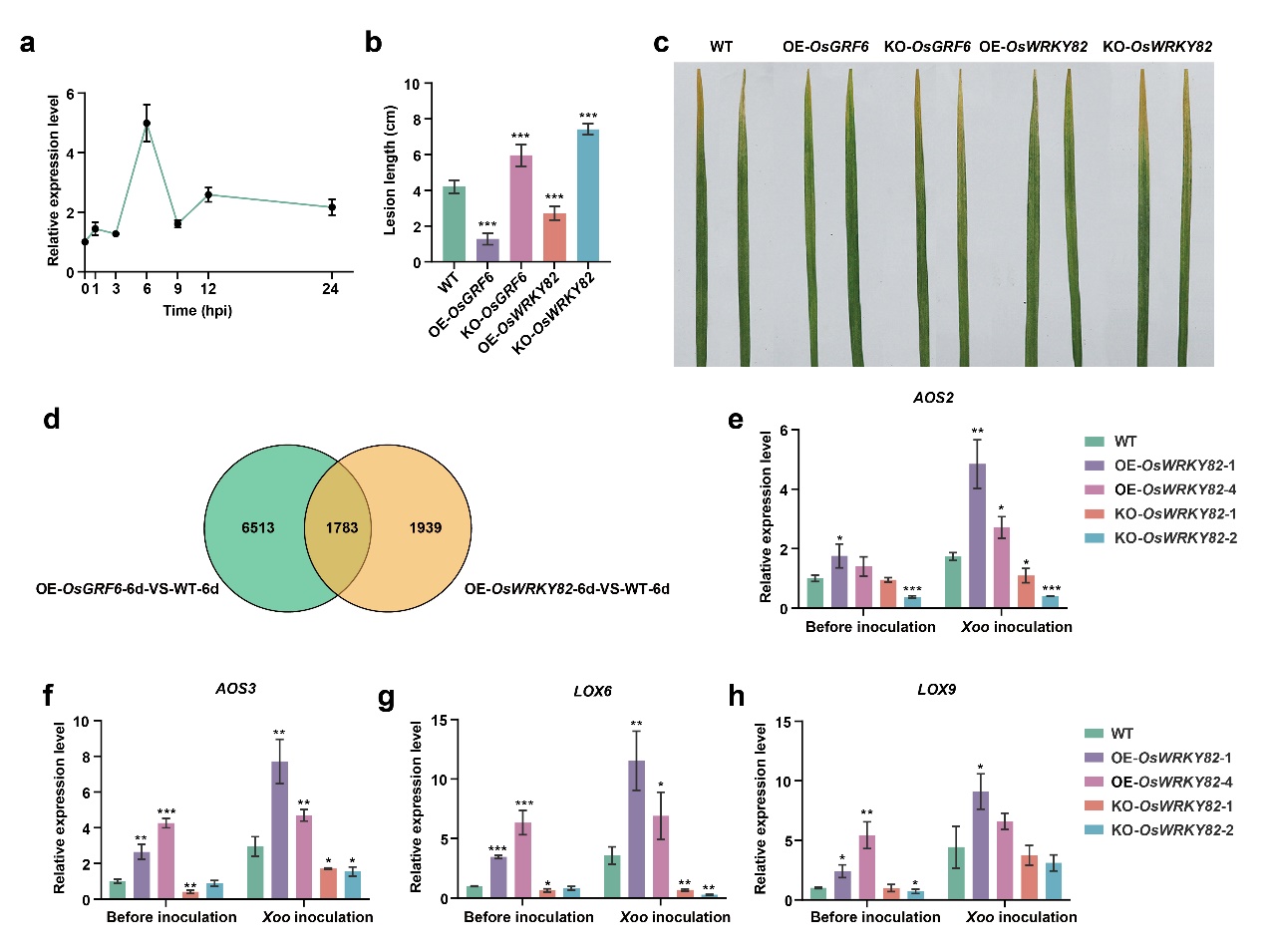


Figure S24. *OsWRKY82* is involved in JA signaling pathway. a) Transcript levels of *OsGRF6* were calculated at 1,3, 6, 9, 12 and 24 h under JA treatment. Data are mean ± s.d. (*n* = 3). b, c) The phenotypes and lesion lengths of *Xoo* inoculated with *OsGRF6* and *OsWRKY82* transgenic lines under JA treatment. The 30-day-old leaves were inoculated with the *Xoo* and the leaves were photographed and measured at 14 dpi. Data are mean ± s.d. (*n* = 5). d) Venn diagrams of the DEGs in *Xoo* inoculated OE-*OsGRF6* and OE-*OsWRKY82* lines. e-h) The expression level of *AOS2* (e), *AOS2* (f), *LOX6* (g) and *LOX9* (h) in *OsWRKY82* transgenic lines after *Xoo* infection for 6 days. Data are mean ± s.d. (*n* = 3). Different asterisks in a, b, e, f, g and h indicate significant differences determined by Student’s *t-*test (*, *P* < 0.05; **, *P* < 0.01; ***, *P* < 0.001).


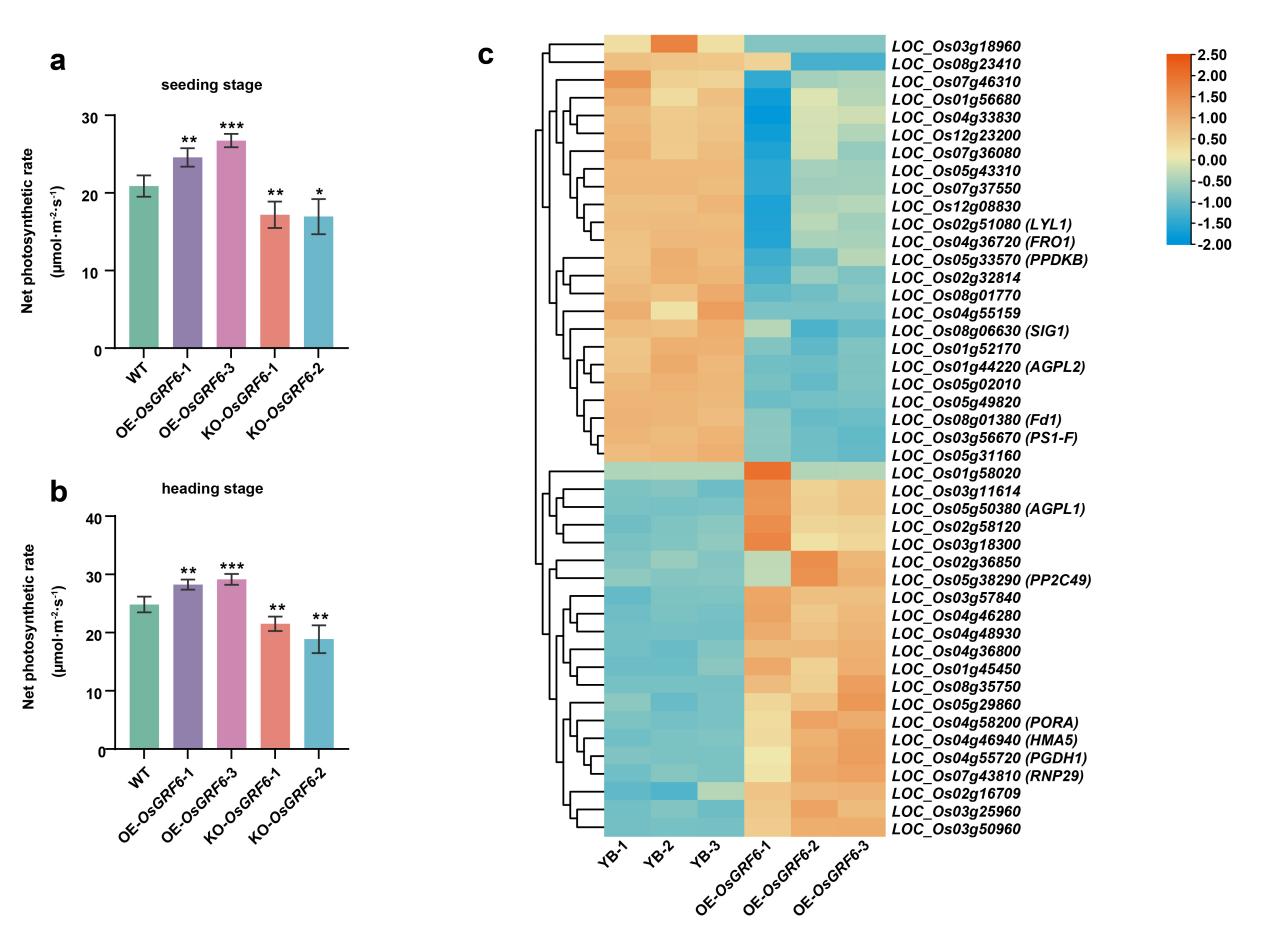


Figure S25. *OsGRF6* enhance photosynthetic efficiency in rice. a, b) Statistical analysis of the photosynthetic efficiency of *OsGRF6* transgenic lines at seedling stage (a) and heading stage (b), respectively. c) Heatmaps of DEGs clustering to photosynthesis signaling pathway in YB VS OE-*OsGRF6* rice seedlings. Different asterisks indicate significant differences determined by Student’s *t-*test (*, *P* < 0.05; **, *P* < 0.01; ***, *P* < 0.001). Data are mean ± s.d. (*n* = 3).


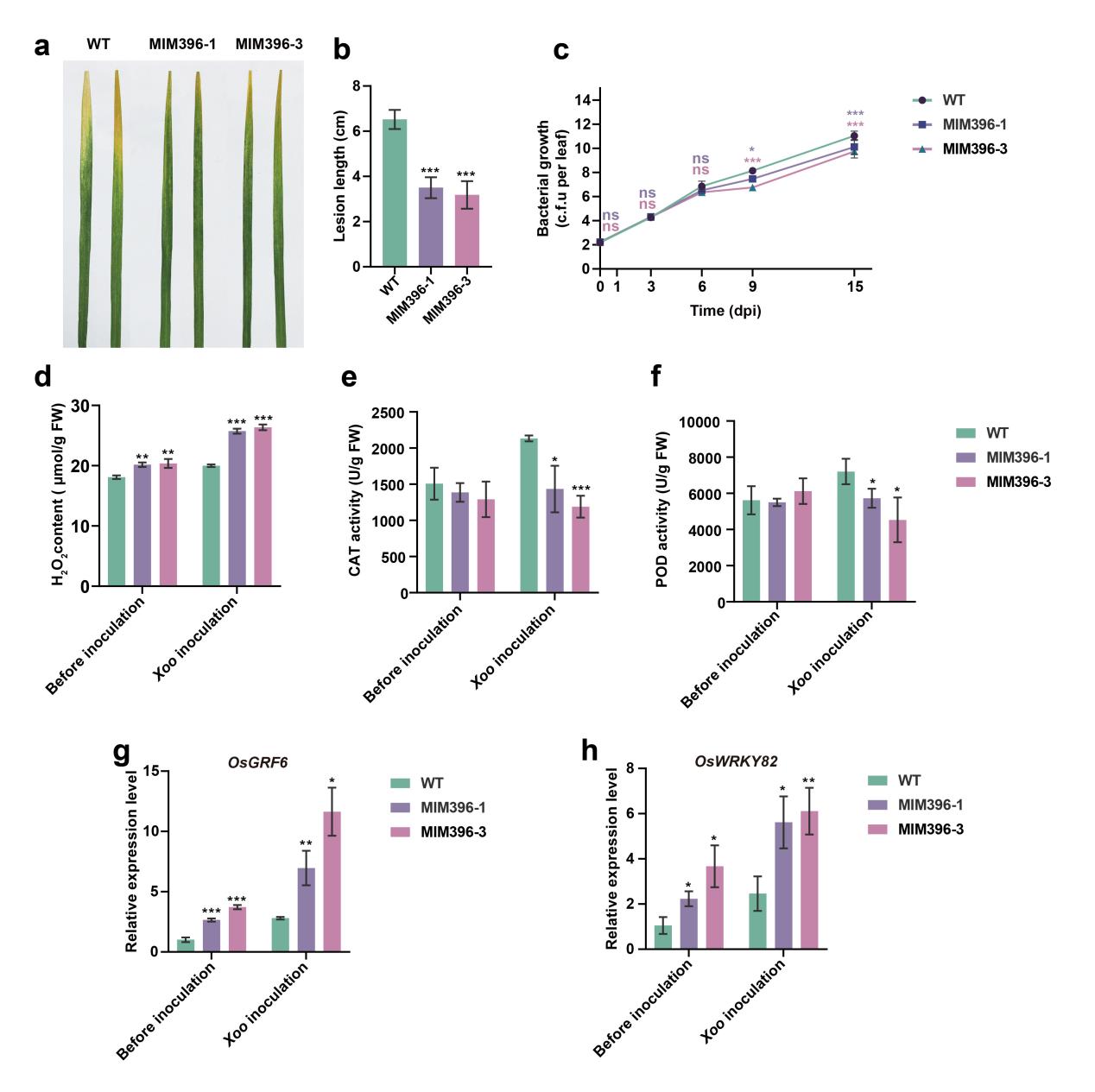


Figure S26. miR396b negatively regulates disease resistance in rice. a, b) The phenotypes and lesion lengths of MIM396 transgenic lines after *Xoo* inoculation. The 30-day-old leaves were inoculated with the *Xoo* and the leaves were photographed and measured at 14 dpi. Data are mean ± s.d. (*n* = 5). c) Growth of *Xoo* in leaves of the MIM396 transgenic lines. The bacterial growth in WT and MIM396 lines was calculated at 0, 3, 6, 9 and 15 dpi compared with the value at 0 day. Data are mean ± s.d. (*n* = 3). d-f) Measurements of H_2_O_2_ (d), CAT (e) and POD (f) in the MIM396 transgenic lines after *Xoo* inoculation for 6 days. Data are mean ± s.d. (*n* = 3). g, h) Expression level of *OsGRF6* (g) and *OsWRKY82* (h) in the WT and MIM396 transgenic lines after *Xoo* infection for 6 days. Data are mean ± s.d. (*n* = 3). Different asterisks in b, c, d, e, f, g and h indicate significant differences determined by Student’s *t-*test (*, *P* < 0.05; **, *P* < 0.01; ***, *P* < 0.001).


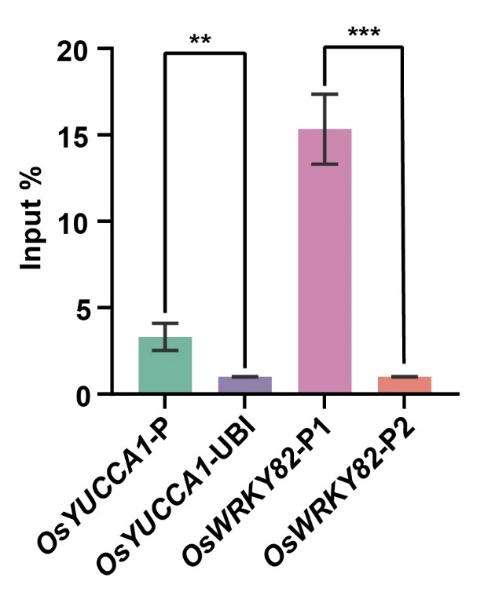


Figure S27. ChIP-qPCR analysis of *OsYUCCA1* and *OsWRKY82* binding efficiency in OE-*OsGRF6* lines after *Xoo* inoculation. The 30-day-old leaves were inoculated with the *Xoo* for 6 days and collected for ChIP-qPCR analysis. "P" and "UBI" represent the OsGRF6 binding sites on the *OsYUCCA1* promoter and the corresponding negative control, while "P1" and "P2" represent the OsGRF6 binding sites on the *OsWRKY82* promoter and their respective negative controls. Data are mean ± s.d. (*n* = 3). Different asterisks indicate significant differences determined by Student’s *t-*test (**, *P* < 0.01; ***, *P* < 0.001).


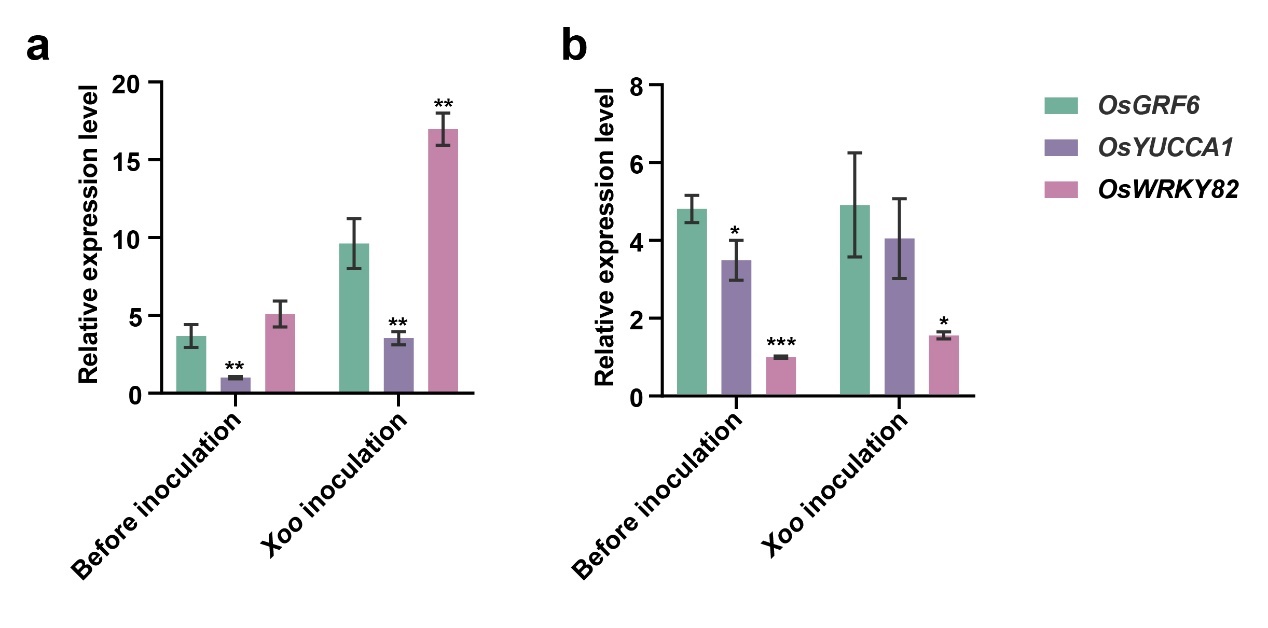


Figure S28. qRT–PCR analysis of the *OsGRF6*, *OsYUCCA1* and *OsWRKY82* expression in leaves and young inflorescence after *Xoo* inoculation. were measured in leaves (a) and young inflorescences (0.5–1 cm) (b) from field-grown rice plants at the booting stage. Different asterisks indicate significant differences determined by Student’s *t-*test (*, *P* < 0.05; **, *P* < 0.01; ***, *P* < 0.001). Data are mean ± s.d. (*n* = 3).

Table S1. The Pi value of *OsGRF6* and its flanking region.

| **IND** | **JAP** | **Wild** | **nuc.MB** |
| --- | --- | --- | --- |
| 0.003798009 | 0.000383422 | 0.000528027 | 29.724449 |
| 0.000233882 | 4.17E-05 | 0.000101798 | 29.726449 |
| 0.001432942 | 8.24E-05 | 9.15E-05 | 29.728449 |
| 0.000791375 | 4.17E-05 | 0.000316499 | 29.730449 |
| 0.003123839 | 0.000478723 | 0.001131676 | 29.732449 |
| 0.003117675 | 0.000262855 | 0.000878371 | 29.734449 |
| 0 | 0 | 0 | 29.736449 |
| 0 | 0 | 0 | 29.738449 |
| 0 | 0 | 0 | 29.740449 |
| 0.003974095 | 0.000537677 | 0.001145161 | 29.742449 |
| 0.002378297 | 0.0001875 | 0.00065706 | 29.744449 |
| 0.000177531 | 4.08E-05 | 0 | 29.746449 |
| 0.005935232 | 0.001222518 | 0.001024855 | 29.748449 |
| 0.005491143 | 0.000823138 | 0.001532787 | 29.750449 |
| 0.002994657 | 0.000342642 | 0.000855368 | 29.752449 |
| 0.004691727 | 0.001046543 | 0.001141988 | 29.754449 |
| 0.005444247 | 0.000777926 | 0.001696721 | 29.756449 |
| 0.000889289 | 4.08E-05 | 0.000185087 | 29.758449 |
| 0.001626341 | 0.000253989 | 0.000488366 | 29.760449 |
| 0.003390707 | 0.000636525 | 0.000911423 | 29.762449 |
| 0.008620674 | 0.000949025 | 0.002043892 | 29.764449 |
| 0.002662738 | 0.000507092 | 0.001283184 | 29.766449 |
| 0.000886706 | 0.000196809 | 0 | 29.768449 |
| 0.001538078 | 0.000182181 | 0.000316764 | 29.770449 |
| 0.001110552 | 0.000139628 | 0.000195135 | 29.772449 |
| 0.000934643 | 0.000419326 | 0.000280011 | 29.774449 |
| 0.000785805 | 0.000100621 | 0.000565309 | 29.776449 |
| 0.001802521 | 0.000304521 | 0.000247488 | 29.778449 |
| 0.008428351 | 0.002158245 | 0.002631941 | 29.780449 |
| 0.002659792 | 0.000641401 | 0.000846906 | 29.782449 |
| 0.001332294 | 0.000263741 | 0.000246959 | 29.784449 |
| 0.001218533 | 0.000164894 | 0.000480962 | 29.786449 |
| 0.003716291 | 0.000699911 | 0.000991539 | 29.788449 |
| 0.000739028 | 0.00010328 | 0 | 29.790449 |
| 0.002949348 | 0.001161791 | 0.001135114 | 29.792449 |
| 0.001361451 | 0.000429078 | 0.000195399 | 29.794449 |
| 0.000301865 | 4.17E-05 | 6.13E-05 | 29.796449 |
| 0.001494368 | 0.000342642 | 0.000125331 | 29.798449 |
| 0.002903827 | 0.001616135 | 0.000332893 | 29.800449 |
| 0.001944319 | 0.000243794 | 0.000611581 | 29.802449 |
| 0.004450648 | 0.001167553 | 0.001038075 | 29.804449 |
| 0.00134981 | 0.000933954 | 0.000471708 | 29.806449 |
| 0.002757438 | 0.000721631 | 0.000954257 | 29.808449 |
| 0.003980628 | 0.001640071 | 0.001283712 | 29.810449 |
| 0.008195809 | 0.001527926 | 0.002911687 | 29.812449 |
| 0.004481671 | 0.001130319 | 0.002257007 | 29.814449 |
| 0.000780591 | 0.000414007 | 0.00019064 | 29.816449 |
| 0.001665186 | 0.000766401 | 0.000177155 | 29.818449 |
| 0.002554814 | 0.000574025 | 0.000387097 | 29.820449 |
| 0.00025028 | 2.08E-05 | 0 | 29.822449 |
| 0.000279106 | 6.16E-05 | 7.54E-05 | 29.824449 |
| 0.000249909 | 0 | 0 | 29.826449 |
| 0.000537526 | 0.000158688 | 0 | 29.828449 |
| 0.000295889 | 6.16E-05 | 0.000227922 | 29.830449 |
| 0.001072727 | 0.001022606 | 0.000536224 | 29.832449 |
| 0.003143985 | 0.000839096 | 0.000848228 | 29.834449 |
| 0.000227013 | 4.08E-05 | 0 | 29.836449 |
| 0.001142706 | 0.00014406 | 0.000114225 | 29.838449 |
| 0.000554266 | 0.000140514 | 0.000209413 | 29.840449 |
| 0.000564711 | 8.33E-05 | 0 | 29.842449 |
| 0.003783583 | 0.00058289 | 0.00079799 | 29.844449 |
| 0.001551939 | 0.000485372 | 0 | 29.846449 |
| 0.002649357 | 0.000925975 | 0.000603913 | 29.848449 |
| 0.000378994 | 0.000405585 | 0.000142253 | 29.850449 |
| 0.002687288 | 0.000558954 | 0.000572448 | 29.852449 |
| 0.002758835 | 0.000590869 | 0.000710999 | 29.854449 |
| 0.002557083 | 0.00075266 | 0.001392121 | 29.856449 |
| 0.003370797 | 0.000957004 | 0.000382073 | 29.858449 |
| 0.001840159 | 0.00158023 | 9.31E-05 | 29.860449 |
| 0.000472737 | 0.000613475 | 4.79E-05 | 29.862449 |
| 0.001060175 | 0.000488918 | 3.17E-05 | 29.864449 |
| 0.000689376 | 0.000740248 | 0 | 29.866449 |
| 0.000735271 | 0.000898493 | 3.17E-05 | 29.868449 |
| 0.000855825 | 0.000462766 | 0.000306187 | 29.870449 |
| 0.00038535 | 0.000214096 | 0 | 29.872449 |
| 0.000846559 | 0.000300089 | 0.000453993 | 29.874449 |
| 0.001789749 | 0.000849291 | 0.000265732 | 29.876449 |
| 0.000903363 | 0.00062367 | 3.17E-05 | 29.878449 |
| 0.002078233 | 0.00135328 | 0.000843998 | 29.880449 |
| 0.002033545 | 0.000928191 | 0.000424643 | 29.882449 |
| 0.001290845 | 0.000272163 | 0.000222369 | 29.884449 |
| 0.001483146 | 0.000591312 | 0.000480169 | 29.886449 |
| 0.002962338 | 0.000962766 | 0.00033871 | 29.888449 |
| 0.001890271 | 0.000926862 | 0.000524061 | 29.890449 |
| 0.001668768 | 0.000740691 | 0.000180063 | 29.892449 |
| 0.00097657 | 0.000371011 | 6.35E-05 | 29.894449 |
| 0.001288926 | 0.000640514 | 3.17E-05 | 29.896449 |
| 0.00251638 | 0.000485816 | 0.000309096 | 29.898449 |
| 0.000921987 | 0.000329344 | 9.31E-05 | 29.900449 |
| 0.000991742 | 0.000371011 | 4.68E-05 | 29.902449 |
| 0.001485839 | 0.000533688 | 0.000130354 | 29.904449 |
| 0.001204688 | 0.000296543 | 3.17E-05 | 29.906449 |
| 0.001227745 | 0.000563387 | 0.000114225 | 29.908449 |
| 0.000892565 | 0.00055984 | 0.000122686 | 29.910449 |
| 0.003459482 | 0.000549202 | 0.000289001 | 29.912449 |
| 0.00016901 | 2.08E-05 | 0.000114225 | 29.914449 |
| 5.58E-06 | 0 | 0 | 29.916449 |
| 0 | 0 | 0 | 29.918449 |
| 0 | 0 | 0 | 29.920449 |
| 0.00030855 | 9.88E-05 | 1.61E-05 | 29.922449 |
| 0 | 2.08E-05 | 0 | 29.924449 |

Table S2. The Fst value of *OsGRF*6 and its flanking genomic regions.

| **MB** | **haplotype.F*_ST_*** | **nucleotide.F*_ST_*** | **Nei.G*_ST_*** | **Hudson.G*_ST_*** | **Hudson.H*_ST_*** |
| --- | --- | --- | --- | --- | --- |
| 29.724449 | 0.472897954 | 0.425321935 | 0.310439807 | 0.236907811 | 0.236415535 |
| 29.726449 | 0.219117052 | 0.186346301 | 0.12378261 | 0.083385961 | 0.082452971 |
| 29.728449 | 0.546134332 | 0.279456602 | 0.376286623 | 0.279553349 | 0.278970428 |
| 29.730449 | 0.67603403 | 0.397985934 | 0.511168773 | 0.402617816 | 0.402220078 |
| 29.732449 | 0.38417255 | 0.255765416 | 0.238600861 | 0.18233143 | 0.181837274 |
| 29.734449 | 0.443581779 | 0.359709487 | 0.285788576 | 0.216864146 | 0.216350434 |
| 29.736449 | 0 | 0 | 0 | 0 | 0 |
| 29.738449 | 0 | 0 | 0 | 0 | 0 |
| 29.740449 | 0 | 0 | 0 | 0 | 0 |
| 29.742449 | 0.438978669 | 0.407896685 | 0.281986108 | 0.211745395 | 0.211195 |
| 29.744449 | 0.410415774 | 0.28431318 | 0.258870106 | 0.181238686 | 0.180452018 |
| 29.746449 | 0.1999317 | 0.199693179 | 0.111802564 | 0.07436739 | 0.073392773 |
| 29.748449 | 0.239679677 | 0.448576463 | 0.137212019 | 0.115052096 | 0.114886658 |
| 29.750449 | 0.291239115 | 0.334592125 | 0.171457424 | 0.142187364 | 0.141984197 |
| 29.752449 | 0.538650823 | 0.406801516 | 0.36925942 | 0.275610253 | 0.275043089 |
| 29.754449 | 0.260172628 | 0.516577736 | 0.150580934 | 0.125746191 | 0.125566728 |
| 29.756449 | 0.43471574 | 0.464117101 | 0.27856572 | 0.217796869 | 0.217388198 |
| 29.758449 | 0.562749892 | 0.295362337 | 0.392172334 | 0.291913688 | 0.29133693 |
| 29.760449 | 0.417552817 | 0.324904701 | 0.264665232 | 0.199767983 | 0.199233741 |
| 29.762449 | 0.455344981 | 0.541801664 | 0.295571339 | 0.22528943 | 0.224792015 |
| 29.764449 | 0.402080718 | 0.392675782 | 0.252478238 | 0.195240453 | 0.194789976 |
| 29.766449 | 0.490843699 | 0.455750752 | 0.325990453 | 0.247975019 | 0.247472237 |
| 29.768449 | 0.516622103 | 0.538244433 | 0.349026615 | 0.270702234 | 0.270267871 |
| 29.770449 | 0.55504793 | 0.323651404 | 0.384774092 | 0.28787057 | 0.287311234 |
| 29.772449 | 0.58276064 | 0.403392389 | 0.411815077 | 0.310198537 | 0.30966114 |
| 29.774449 | 0.335130508 | 0.52503469 | 0.202333487 | 0.172888454 | 0.172805853 |
| 29.776449 | 0.634316955 | 0.410355483 | 0.465065925 | 0.360562076 | 0.360118109 |
| 29.778449 | 0.563845165 | 0.491209017 | 0.393366639 | 0.316299176 | 0.315992193 |
| 29.780449 | 0.256049737 | 0.609707785 | 0.147807257 | 0.118300905 | 0.117960258 |
| 29.782449 | 0.409274859 | 0.52223341 | 0.258224094 | 0.211890854 | 0.211658195 |
| 29.784449 | 0.587888863 | 0.493601851 | 0.417029707 | 0.331787795 | 0.331440533 |
| 29.786449 | 0.302112921 | 0.336743169 | 0.178670861 | 0.123348934 | 0.122504108 |
| 29.788449 | 0.414631797 | 0.538403787 | 0.262397818 | 0.205534522 | 0.205131042 |
| 29.790449 | 0.304548832 | 0.310380931 | 0.180363503 | 0.124654377 | 0.123813385 |
| 29.792449 | 0.342554491 | 0.719025019 | 0.207698291 | 0.175908649 | 0.175792002 |
| 29.794449 | 0.517155029 | 0.557199146 | 0.349586778 | 0.283992239 | 0.283724956 |
| 29.796449 | 0.49529735 | 0.455340838 | 0.329823149 | 0.238854961 | 0.238185574 |
| 29.798449 | 0.348232267 | 0.422936659 | 0.211766592 | 0.169966766 | 0.16964626 |
| 29.800449 | 0.381210308 | 0.720561541 | 0.236460624 | 0.196051839 | 0.195856367 |
| 29.802449 | 0.512417843 | 0.497265733 | 0.345156732 | 0.257890874 | 0.257327026 |
| 29.804449 | 0.277089595 | 0.554821859 | 0.161852582 | 0.134329249 | 0.13412771 |
| 29.806449 | 0.381677561 | 0.722864947 | 0.236862307 | 0.202745037 | 0.202673059 |
| 29.808449 | 0.457654492 | 0.519301477 | 0.297603484 | 0.240786715 | 0.240503973 |
| 29.810449 | 0.283361603 | 0.687513314 | 0.166099359 | 0.138700849 | 0.138523531 |
| 29.812449 | 0.214764214 | 0.575598636 | 0.121304411 | 0.097201349 | 0.096861236 |
| 29.814449 | 0.471790229 | 0.586141561 | 0.309604814 | 0.253573918 | 0.253336979 |
| 29.816449 | 0.493777279 | 0.703692227 | 0.328705037 | 0.27201203 | 0.271813977 |
| 29.818449 | 0.378608915 | 0.711073191 | 0.234477369 | 0.194052292 | 0.193849643 |
| 29.820449 | 0.490049534 | 0.570511845 | 0.325335349 | 0.253663414 | 0.253247421 |
| 29.822449 | 0.48465961 | 0.48465961 | 0.320473644 | 0.228204653 | 0.227479273 |
| 29.824449 | 0.316780463 | 0.276847815 | 0.18892036 | 0.130001778 | 0.129148496 |
| 29.826449 | 0.520336606 | 0.520336606 | 0.352272727 | 0.253042909 | 0.252347655 |
| 29.828449 | 0.517801588 | 0.702562472 | 0.350215788 | 0.292013067 | 0.291843014 |
| 29.830449 | 0.328093394 | 0.330107391 | 0.196949103 | 0.135246454 | 0.134388058 |
| 29.832449 | 0.326153187 | 0.762281009 | 0.195932095 | 0.172120813 | 0.172148085 |
| 29.834449 | 0.406729895 | 0.645798825 | 0.256141365 | 0.199896176 | 0.199478828 |
| 29.836449 | 0.318604752 | 0.318604752 | 0.190205429 | 0.130661495 | 0.129802985 |
| 29.838449 | 0.670624937 | 0.391665284 | 0.505062074 | 0.404419517 | 0.404080645 |
| 29.840449 | 0.630994804 | 0.62368743 | 0.461574011 | 0.369337588 | 0.369004789 |
| 29.842449 | 0.3157828 | 0.279566206 | 0.188206662 | 0.128702904 | 0.127829619 |
| 29.844449 | 0.41177114 | 0.510423919 | 0.260114747 | 0.202058751 | 0.20162523 |
| 29.846449 | 0.495324281 | 0.588717741 | 0.330059063 | 0.271436742 | 0.271215307 |
| 29.848449 | 0.407034022 | 0.728294981 | 0.256446525 | 0.208950776 | 0.208691437 |
| 29.850449 | 0.55261301 | 0.800528229 | 0.38265022 | 0.322680682 | 0.322551181 |
| 29.852449 | 0.471427546 | 0.569815009 | 0.309215256 | 0.240891711 | 0.240472124 |
| 29.854449 | 0.530943963 | 0.47326139 | 0.362212121 | 0.29066962 | 0.290357151 |
| 29.856449 | 0.313128119 | 0.681151089 | 0.186640034 | 0.155382261 | 0.155196271 |
| 29.858449 | 0.36630318 | 0.669421357 | 0.225233069 | 0.191690266 | 0.191594665 |
| 29.860449 | 0.432893166 | 0.849173784 | 0.277170168 | 0.229637413 | 0.229442507 |
| 29.862449 | 0.514593122 | 0.858103196 | 0.347328441 | 0.294132101 | 0.294021229 |
| 29.864449 | 0.470466698 | 0.759016593 | 0.308521407 | 0.260773278 | 0.260655869 |
| 29.866449 | 0.429971616 | 0.820932346 | 0.274844881 | 0.235201228 | 0.235130972 |
| 29.868449 | 0.316108902 | 0.803671989 | 0.188821007 | 0.167337264 | 0.167399426 |
| 29.870449 | 0.404297379 | 0.775368242 | 0.254339557 | 0.213594922 | 0.213450227 |
| 29.872449 | 0.604532011 | 0.751198275 | 0.433964911 | 0.358877007 | 0.358667959 |
| 29.874449 | 0.591031239 | 0.720642505 | 0.420254526 | 0.348541844 | 0.348345467 |
| 29.876449 | 0.48644805 | 0.708594578 | 0.32227944 | 0.266270725 | 0.266066703 |
| 29.878449 | 0.495424023 | 0.793768327 | 0.330151384 | 0.272261351 | 0.272050231 |
| 29.880449 | 0.32441791 | 0.68288204 | 0.194711419 | 0.173145692 | 0.173222483 |
| 29.882449 | 0.331608717 | 0.696912675 | 0.199765405 | 0.166517275 | 0.166337908 |
| 29.884449 | 0.628522933 | 0.479148145 | 0.45898888 | 0.376712767 | 0.376471755 |
| 29.886449 | 0.57978803 | 0.751475771 | 0.409070046 | 0.347447476 | 0.347342142 |
| 29.888449 | 0.544267926 | 0.587967809 | 0.374744184 | 0.317232765 | 0.317118645 |
| 29.890449 | 0.515235939 | 0.683253738 | 0.347928898 | 0.298159818 | 0.298094724 |
| 29.892449 | 0.575376646 | 0.681833239 | 0.404707215 | 0.342135852 | 0.342013339 |
| 29.894449 | 0.58695507 | 0.636372646 | 0.416161828 | 0.344479165 | 0.344276152 |
| 29.896449 | 0.499530612 | 0.759200722 | 0.333833464 | 0.283997937 | 0.283905176 |
| 29.898449 | 0.551929884 | 0.569196116 | 0.381960426 | 0.314418731 | 0.314198684 |
| 29.900449 | 0.619114331 | 0.570867252 | 0.449099918 | 0.375923419 | 0.375756648 |
| 29.902449 | 0.55592297 | 0.630439935 | 0.385790865 | 0.320713383 | 0.320529713 |
| 29.904449 | 0.516078791 | 0.665485058 | 0.34863074 | 0.286982524 | 0.286764293 |
| 29.906449 | 0.591539182 | 0.527356545 | 0.420735191 | 0.342610103 | 0.342346331 |
| 29.908449 | 0.452532093 | 0.620888182 | 0.293411859 | 0.253202834 | 0.253166084 |
| 29.910449 | 0.50311216 | 0.807707901 | 0.336966031 | 0.27694011 | 0.276716216 |
| 29.912449 | 0.513122055 | 0.473385099 | 0.345938462 | 0.282069964 | 0.281816434 |
| 29.914449 | 0.172363984 | 0.162974429 | 0.095049166 | 0.062904297 | 0.061911349 |
| 29.916449 | 0.004207574 | 0.004207574 | 0.002808989 | 0.001475071 | 0.000263305 |
| 29.918449 | 0 | 0 | 0 | 0 | 0 |
| 29.920449 | 0 | 0 | 0 | 0 | 0 |
| 29.922449 | 0.708152249 | 0.737697369 | 0.548717239 | 0.444406682 | 0.444096287 |
| 29.924449 | 0.00619195 | 0.00619195 | 0.004651163 | 0.004424431 | 0.00658985 |

Table S3. Primers and probes used in this study.

| **Primers** | **Primer sequence** |
| --- | --- |
| **For transgenic assay** |  |
| YUCCA1-gRT1 | TACCCGACGTACCCGTCCAgttttagagctagaaat |
| YUCCA1-OsU6aT1 | TGGACGGGTACGTCGGGTACggcagccaagccagca |
| YUCCA1-gRT2 | CCGGCATGCAGAAGTTCGCCgttttagagctagaaat |
| YUCCA1-OsU6aT2 | GGCGAACTTCTGCATGCCGGCaacacaagcggcagc |
| YUCCA1-OE-F | CTGCAGGTCGACTCTAGAGGATCCATGGACAACAAGCCGGCGCA |
| YUCCA1-OE-R | AGAGATGAGTTTCTGCTCGGATCCAAAGGAAGAGTTGCTTATTT |
| WRKY82-gRT1 | CGCGAATCTTCCATGCCCGAgttttagagctagaaat |
| WRKY82-OsU6aT1 | CAGGGCGCTCCATCCCGGGgttttagagctagaaat |
| WRKY82-gRT2 | TCGGGCATGGAAGATTCGCGCggcagccaagccagca |
| WRKY82-OsU6aT2 | CCCGGGATGGAGCGCCCTGCaacacaagcggcagc |
| WRKY82-OE-F | CTGCAGGTCGACTCTAGAGGATCCATGCCCGACGGCTATCCGGCG |
| WRKY82-OE-R | AGAGATGAGTTTCTGCTCGGATCCATTATTTTGGACAGGAACTAC |
| **For qRT-PCR assay** |  |
| Ubq-F | GCTCCGTGGCGGTATCAT |
| Ubq-R | CGGCAGTTGACAGCCCTAG |
| GRF6-qRT-PCR-F | TCATCAGCCTCAACATCTCC |
| GRF6-qRT-PCR-R | CATCCAGTTTGCTTCTCCCT |
| PR1b-qRT-PCR-F | GGCAACTTCGTCGGACAGA |
| PR1b-qRT-PCR-R | CCGTGGACCTGTTTACATTTTCA |
| PR4-qRT-PCR-F | AGCGCATATTGTGCCACATG |
| PR4-qRT-PCR-R | GGATACACTTGCCACACGAGTCT |
| PR5-qRT-PCR-F | CAACAGCAACTACCAAGTCGTC |
| PR5-qRT-PCR-R | CAAGGTGTCGTTTTATTCATCAAC |
| PR10-qRT-PCR-F | CCCTGCCGAATACGCCTAA |
| PR10-qRT-PCR-R | CTCAAACGCCACGAGAATTTG |
| LOC_Os01g03914-qRT-PCR-F | GAGTTGAGGCAAATGGCAAAG |
| LOC_Os01g03914-qRT-PCR-R | ATCCAGTAGGGAGTCCAGTGTC |
| LOC_Os01g16870-qRT-PCR-F | GACAAACTCCAGCAGACCTA |
| LOC_Os01g16870-qRT-PCR-R | CCAGAACTACCGTGAACTCAT |
| LOC_Os01g22900-qRT-PCR-F | AGACGCTCAACTACGACCAGG |
| LOC_Os01g22900-qRT-PCR-R | TGTAACAATCAACAGTCTTCTCCC |
| LOC_Os02g32350-qRT-PCR-F | TGTTTACCTCGGTGACAAGA |
| LOC_Os02g32350-qRT-PCR-R | AGCAACTTCCTCCAACCTTA |
| LOC_Os02g49140-qRT-PCR-F | CGCCCGTTCGTGACGCACTT |
| LOC_Os02g49140-qRT-PCR-R | ATGCCCTCGTCGCAGCTCTTCC |
| LOC_Os02g52270-qRT-PCR-F | CGACTTCATCTCCCTCCTCT |
| LOC_Os02g52270-qRT-PCR-R | TTCTTCGCCCTGCTTCTTGT |
| LOC_Os03g29850-qRT-PCR-F | AGACTTTCGCCGACCTCCTCCC |
| LOC_Os03g29850-qRT-PCR-R | TGCTGCTCAGCTTACCCTCCTCC |
| LOC_Os04g56100-qRT-PCR-F | CGGCCGCGGTGGTGATCTTG |
| LOC_Os04g56100-qRT-PCR-R | CTGTTCTTCCGCCGCTGCCT |
| LOC_Os04g57760-qRT-PCR-F | CGACGCCTGCAACGGGATCA |
| LOC_Os04g57760-qRT-PCR-R | AGCGGTGGTGACGACGACGAG |
| LOC_Os06g23290-qRT-PCR-F | GCTGGCAGTGGAGGGAGATG |
| LOC_Os06g23290-qRT-PCR-R | CTGGCTTGCGGATGAACAAA |
| LOC_Os06g44160-qRT-PCR-F | AGAGCGATAGCGGCGTCCAT |
| LOC_Os06g44160-qRT-PCR-R | TCGTCTTCGTCGTTGAGGTGGT |
| LOC_Os06g44180-qRT-PCR-F | GACTCCACCGGCTTCAAGGAC |
| LOC_Os06g44180-qRT-PCR-R | GACAGCAGCTTCGACAGGATGTAC |
| LOC_Os07g37454-qRT-PCR-F | ACGGCGTTCAACATCATCCTCA |
| LOC_Os07g37454-qRT-PCR-R | CGAACACCCCGAACGACCAGA |
| LOC_Os07g43800-qRT-PCR-F | GAGCCGCTGCTGCTGATCCCTT |
| LOC_Os07g43800-qRT-PCR-R | AATCCGCCGCCGCCGACAAGTA |
| LOC_Os10g35990-qRT-PCR-F | AAAGGCAACGAGTTTGAGGA |
| LOC_Os10g35990-qRT-PCR-R | CAAGAATATCGCTACCAGTCA |
| WRKY82-qRT-PCR-F | ACGGCTCAGGCACAAATACA |
| WRKY82-qRT-PCR-R | TGCGAACACTCCCTTCAGTT |
| LOX9-qRT-PCR-F | TGCGTCCTCAGCTCAATCCATC |
| LOX9-qRT-PCR-R | CAACAACAACCCGACTGCTACCTT |
| LOX6-qRT-PCR-F | CGTGGCTGCGTGACGACGAGTT |
| LOX6-qRT-PCR-R | CGAGGTGCCCGATGATGTGC |
| AOS3-qRT-PCR-F | GACGACGCCGAGAAGAGCCACA |
| AOS3-qRT-PCR-R | CGGGCGAGCCACTTGACGAT |
| AOS2-qRT-PCR-F | CTCGTCGGAAGGCTGTTGCTC |
| AOS2-qRT-PCR-R | CGATTGACGGCGGAGGTTGA |
| ARF1-qRT-PCR-F | GTGCCGTGAGTAGTTCAGTTT |
| ARF1-qRT-PCR-R | TCTTGCTTCATTGCTGGTAA |
| ARF2-qRT-PCR-F | AGCTGCTTTGCCGTGTCCTC |
| ARF2-qRT-PCR-R | TCTTCTCCACCGCCATCTCA |
| ARF3-qRT-PCR-F | GCGGAGGGCGAGGTATTTGA |
| ARF3-qRT-PCR-R | AGCAGCCCGACGAGGAACAG |
| ARF5-qRT-PCR-F | TAGGATTCCCAATAGAAACAGA |
| ARF5-qRT-PCR-R | AAAGCCATATCCGACTGACC |
| ARF6-qRT-PCR-F | CCAACAGGCTTACCTTCTTT |
| ARF6-qRT-PCR-R | ACTCATACCAAGTCCTCCAAA |
| ARF7-qRT-PCR-F | ATGGGCAAATTCTGACTGGA |
| ARF7-qRT-PCR-R | GTGGCTGAGGTGATGGCTGT |
| ARF8-qRT-PCR-F | CCCGCCACCAAGAATCAGCA |
| ARF8-qRT-PCR-R | CTCTGCCCGAACAGCATTAGCC |
| ARF9-qRT-PCR-F | TTATGCAAGGTATGGCAGTT |
| ARF9-qRT-PCR-R | TCATCCTCATCATCGGTGTA |
| ARF10-qRT-PCR-F | TGGTTCATGGGCACTGTCGC |
| ARF10-qRT-PCR-R | GGATGGCAGGTGGAGGTTGG |
| ARF11-qRT-PCR-F | ATTCCCGCATTCCGAACTAC |
| ARF11-qRT-PCR-R | TGGCTGAAGGGTCATCTGTG |
| ARF12-qRT-PCR-F | ATCAAGCGTTCGCAGGTATA |
| ARF12-qRT-PCR-R | CATAAAGAAACTCTTGGTGGTCT |
| ARF13-qRT-PCR-F | GGGTGGAGGTGGATAAGGGAT |
| ARF13-qRT-PCR-R | AGGTTAGTGAGGGTGCCATTGAG |
| ARF14-qRT-PCR-F | TCCCTCGTCGGTTCTGATGT |
| ARF14-qRT-PCR-R | GTGATTTCGCTCGGCGTTTG |
| ARF15-qRT-PCR-F | AGCACTGGCTCTTCCATCTC |
| ARF15-qRT-PCR-R | AAACCTAACACGCCTATCATTCT |
| ARF16-qRT-PCR-F | CAGGTCTGAGCCTAGTCCAA |
| ARF16-qRT-PCR-R | CCTCGTTGCTACTACCCATC |
| ARF17-qRT-PCR-F | GGGTAGCCGAGTGGTTTATT |
| ARF17-qRT-PCR-R | AGTTGTGGAGGCAAGTTAGG |
| ARF18-qRT-PCR-F | CTTGGGTGATGCTGCTTCGG |
| ARF18-qRT-PCR-R | TGTGGTGCCATTCTGATTACTTCT |
| ARF19-qRT-PCR-F | ACAATAGCCCATTTACCATCT |
| ARF19-qRT-PCR-R | TTCCCATGTATCTTCGTGTT |
| ARF20-qRT-PCR-F | CAGCAACTACTTCGGCTGTGCG |
| ARF20-qRT-PCR-R | AAGAGGGAAATCATGGCGTAGGAA |
| ARF21-qRT-PCR-F | GGGATGACAGATGACGAGAC |
| ARF21-qRT-PCR-R | GCTAGATTTAGACCAGGCAC |
| ARF22-qRT-PCR-F | CATTGATTTGTCTGTCTTTGGGTC |
| ARF22-qRT-PCR-R | ATTATTGTAAGCCTCCGTGCC |
| ARF23-qRT-PCR-F | ACCACTGGATATGAGCCGTCAG |
| ARF23-qRT-PCR-R | AACAAGCCGTTTGGCACTAA |
| ARF24-qRT-PCR-F | GGGGCTCCGCTCTACAATCT |
| ARF24-qRT-PCR-R | TGGCACCACCTCTTCCTCCT |
| ARF25-qRT-PCR-F | GCCACAAATAGTCGCTTCAC |
| ARF25-qRT-PCR-R | AACGCATTCCAACAGATACA |
| YUCCA1-qRT-PCR-F | CTCACGGCGGCTCACCTCAT |
| YUCCA1-qRT-PCR-R | CCTTGCCTCGTCATCTCCTTCAC |
| YUCCA2-qRT-PCR-F | GCCTGAAGGAGAAGGGAATC |
| YUCCA2-qRT-PCR-R | CACAGAACTGACGAGGTAGATG |
| YUCCA3-qRT-PCR-F | ACTACCCTATTTATCCCTCAAAGC |
| YUCCA3-qRT-PCR-R | ACAACCAACCACCGAGACAC |
| YUCCA4-qRT-PCR-F | GGAGTTTGTGGATGGCAGCAC |
| YUCCA4-qRT-PCR-R | GCCTTGAGAAACCAACAGCGTA |
| YUCCA5-qRT-PCR-F | GCCTCCGACGACCTAAGATTGG |
| YUCCA5-qRT-PCR-R | GGCATCAAAGTCCTCCCTACAGC |
| YUCCA6-qRT-PCR-F | CCCAGTGGCTCAAGGGAAGTGA |
| YUCCA6-qRT-PCR-R | CATGGCAATGTCCTGTGCAACCT |
| YUCCA7-qRT-PCR-F | TGGCTCCAGGGAAATGACTTCTTC |
| YUCCA7-qRT-PCR-R | GTGCTCTTCTTGGTTGGCTTCGTC |
| **For subcellular localization** |  |
| WRKY82-HBT-F | CCTCTCCCCTTGCTCCGTGGATCCATGCCCGACGGCTATCCGGCG |
| WRKY82-HBT-R | CTCGCCCTTGCTCACCATGGATCCATTATTTTGGACAGGAACTAC |
| **For luciferase activity assay** |  |
| WRKY82-LUC-F | TCGAATTCCTGCAGCCCGGGGGATCCACGGTACAAACTATCTCCCT |
| WRKY82-LUC-R | GCGGCCGCTCTAGAACTAGTGGATCCGGAAGATTCGCGGAGGAGGG |
| GAL4DB-WRKY82-F | CCCCCGGGCTGCAGGAATTCATGCCCGACGGCTATCCGGCG |
| GAL4DB-WRKY82-R | ATCGATAAGCTTGATATCGAATTCATTATTTTGGACAGGAACTAC |
| VP16-WRKY82-F | TCCTGCAGGTCGACGGTACCATGCCCGACGGCTATCCGGCG |
| VP16-WRKY82-R | TCCAGCGCGTCCATGGTACCATTATTTTGGACAGGAACTAC |
| YUCCA1-LUC-F | TCGAATTCCTGCAGCCCGGGGGATCCTCTGAAGAGCAATGGAGGTA |
| YUCCA1-LUC-R | GCGGCCGCTCTAGAACTAGTGGATCCCCCTTGTGCAGAGCGGTTGT |
| **For ChIP-PCR** |  |
| UBI-F | ACCACTTCGACCGCCACTACT |
| UBI-R | ACGCCTAAGCCTGCTGGTT |
| YUCCA1-ChIP-PCR-F | ACAAGCGAAATCCTACAGCA |
| YUCCA1-ChIP-PCR-R | ACAAGCGAAATCCTACAGCA |
| WRKY82_Chip-PCR-F | CGCCATCCGACCGTTTCGAC |
| WRKY82_Chip-PCR-R | GGGAGCGGGGAGCCTTTTGA |
| WRKY82_NC_Chip-PCR-F | TCTACGATACAAACTATCTC |
| WRKY82_NC_Chip-PCR-R | ATAGTATGTAGAATGGTGAT |
| **For EMSA** |  |
| EMSA-Probe-F: | TGATGAATTGAAAAGCTTGAATTC-5’FAM |
| EMSA-Probe-R: | GAGTTTTATATACATACAGAGCACATGC-5’FAM |
| MBP-GRF6-F | CGAGGGAAGGATTTCAGAATTCATGCAGGGTGCAATGGCCAG |
| MBP-GRF6-R | CAGGTCGACTCTAGAGGATCCCACCAGGCGGATGCTCGGAT |
| **For yeast one-hybrid assays** |  |
| WRKY82-AbAi-F | AAGCTTGAATTCGAGCTCGGTACCATGCCACATCATCATCCACT |
| WRKY82-AbAi-R | TACATACAGAGCACATGCCTCGAGGGAAGATTCGCGGAGGAGGG |
| YUCCA-AbAi-F | AAGCTTGAATTCGAGCTCGGTACCTGTGTTTAGTGCCATGCAAGTA |
| YUCCA-AbAi-R | TACATACAGAGCACATGCCTCGAGCCCTTGTGCAGAGCGGTTGT |
